# Unraveling the role of Epac1‐SOCS3 signaling in the development of neonatal‐CRD‐induced visceral hypersensitivity in rats

**DOI:** 10.1111/cns.13880

**Published:** 2022-06-15

**Authors:** Si‐Ting Huang, Bin‐Bin Chen, Zhi‐Jing Song, Hui‐Li Tang, Rong Hua, Yong‐Mei Zhang

**Affiliations:** ^1^ Jiangsu Province Key Laboratory of Anesthesiology Xuzhou Medical University Xuzhou China; ^2^ Jiangsu Province Key Laboratory of Anesthesia and Analgesia Application Technology Xuzhou Medical University Xuzhou China; ^3^ NMPA Key Laboratory for Research and Evaluation of Narcotic and Psychotropic Drugs Xuzhou China; ^4^ Department of Emergency The Affiliated Hospital of Xuzhou Medical University Xuzhou China

**Keywords:** corticotrophin‐releasing factor, Epac1, neonatal colorectal distension, SOCS3, visceral hypersensitivity

## Abstract

**Aims:**

Visceral hypersensitivity in irritable bowel syndrome (IBS) is widespread, but effective therapies for it remain elusive. As a canonical anti‐inflammatory protein, suppressor of cytokine signaling 3 (SOCS3) reportedly relays exchange protein 1 directly activated by cAMP (Epac1) signaling and inhibits the intracellular response to inflammatory cytokines. Despite the inhibitory effect of SOCS3 on the pro‐inflammatory response and neuroinflammation in PVN, the systematic investigation of Epac1‐SOCS3 signaling involved in visceral hypersensitivity remains unknown. This study aimed to explore Epac1‐SOCS3 signaling in the activity of hypothalamic paraventricular nucleus (PVN) corticotropin‐releasing factor (CRF) neurons and visceral hypersensitivity in adult rats experiencing neonatal colorectal distension (CRD).

**Methods:**

Rats were subjected to neonatal CRD to simulate visceral hypersensitivity to investigate the effect of Epac1‐SOCS3 signaling on PVN CRF neurons. The expression and activity of Epac1 and SOCS3 in nociceptive hypersensitivity were determined by western blot, RT‐PCR, immunofluorescence, radioimmunoassay, electrophysiology, and pharmacology.

**Results:**

In neonatal‐CRD‐induced visceral hypersensitivity model, Epac1 and SOCS3 expressions were downregulated and IL‐6 levels elevated in PVN. However, infusion of Epac agonist 8‐pCPT in PVN reduced CRF neuronal firing rates, and overexpression of SOCS3 in PVN by AAV‐SOCS3 inhibited the activation of PVN neurons, reduced visceral hypersensitivity, and precluded pain precipitation. Intervention with IL‐6 neutralizing antibody also alleviated the visceral hypersensitivity. In naïve rats, Epac antagonist ESI‐09 in PVN increased CRF neuronal firing. Consistently, genetic knockdown of Epac1 or SOCS3 in PVN potentiated the firing rate of CRF neurons, functionality of HPA axis, and sensitivity of visceral nociception. Moreover, pharmacological intervention with exogenous IL‐6 into PVN simulated the visceral hypersensitivity.

**Conclusions:**

Inactivation of Epac1‐SOCS3 pathway contributed to the neuroinflammation accompanied by the sensitization of CRF neurons in PVN, precipitating visceral hypersensitivity and pain in rats experiencing neonatal CRD.

## Graphical abstract

This study proposed a notion that early‐life stress disrupts the Epac1/Rap1 pathway and subsequently SOCS3, which triggers sensitized CRF neurons in PVN and amplified IL‐6 signaling in neuroinflammation and visceral nociception. (A schema of the experiment. In neonatal‐CRD‐induced visceral hypersensitivity, both Epac1 and SOCS3 were downregulated, resulting in the disinhibition of IL‐6 receptor‐mediated signaling and amplification of IL‐6 pro‐inflammatory signals and further activation of CRF neurons in PVN and the HPA axis. Activation of either Epac1 or SOCS3, or antagonism on IL‐6, reversed the visceral nociceptive effects in the neonatal‐CRD group).

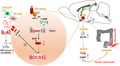

## 1 INTRODUCTION

Irritable bowel syndrome (IBS) is a common disorder in gastroenterology, characterized by aberrant bowel movement, abdominal pain, and distension. A number of patients are complicated with psychological disorders. Despite the high incidence of IBS,^1^ the underlying mechanisms remain elusive. Visceral hypersensitivity, a hallmark of IBS and other diseases with visceral pain,^2^, ^3^ is a multifactorial process in the etiology of IBS.^4^, ^5^, ^6^, ^7^ As the animal model of chronic visceral hypersensitivity described by Al‐Chaer and collaborators, neonatal colorectal distension (CRD) induces adult visceral hypersensitivity long after the initial damage subsides. Recognition of the mechanism of neonatal‐CRD‐induced visceral hypersensitivity is essential to the understanding of IBS and other functional visceral pain and the development of effective therapeutic approaches.

Despite the distinction of animal from humans, animal models are still employed to simulate human symptoms and explore the etiology of human diseases. Early‐life stress (ELS) models such as neonatal maternal separation, neonatal CRD, and limited nesting paradigm predispose specific visceral hypersensitivity and alter stress responses in adulthood.^7^, ^8^, ^9^, ^10^, ^11^ Microglia, the resident macrophages in the central nervous system, can release a variety of pro‐inflammatory cytokines, including interleukin‐1β (IL‐1β), interleukin‐6 (IL‐6), and tumor necrosis factor α (TNF‐α) to maintain homeostasis and immunity.^12^, ^13^ Our prior studies revealed that CRD can result in an activation of microglia in PVN, leading to sensitization of CRF neurons and activation of the hypothalamo‐pituitary–adrenal (HPA) axis.^14^, ^15^ Hypothalamic PVN CRF peptide stimulates the synthesis and secretion of adrenocorticotropic hormone (ACTH) by the anterior pituitary and is the main mediator of HPA axis during stress. Besides the underlying sensitization of CRF neurons in PVN, microglial activation participates in the etiology of visceral hypersensitivity and pain.^16^, ^17^ The neurobiological substrates and molecular mechanisms that connect CRD, microglial activation, and visceral hypersensitivity remain elusive.

Cyclic AMP is an intracellular signaling messenger that alters pain threshold via two different cAMP‐activated proteins: cAMP‐dependent protein kinase (protein kinase A, PKA) and exchange protein directly activated by cAMP (Epac).^18^ In addition to the canonical PKA pathway, previous studies have demonstrated that Epacs regulate pain.^19^, ^20^, ^21^ Intracellular messenger cAMP and its downstream signaling cascades are involved in pain.^22^ Exchange proteins directly activated by cAMP (Epac) are cAMP effectors. Epac family consist Epac1 and Epac2, both being guanine‐nucleotide‐exchange factors that activate the Ras‐like small G proteins Rap1 and Rap2, respectively.^23^ Functionally, Epacs regulate neuronal excitability, axonal guidance, inflammation regulation, learning, long‐term potentiation, cell migration and adhesion, and energy homeostasis, etc.^21^, ^24^, ^25^, ^26^, ^27^, ^28^ Studies have defined a role for Epac1 in cAMP‐mediated, PKA‐independent transcriptional induction of suppressor of cytokine signaling 3 (SOCS3) gene.^29^, ^30^, ^31^ Activation of Epac can facilitate the accumulation of SOCS3, thereby resulting in the SOCS3‐dependent neuroinflammation suppression via inhibition of IL‐6 signaling. Hence, SOCS3 is an important crosstalk point between the Epac1/Rap1 and the IL‐6/JAK/STAT pathways.^32^ In this study, we explored the role of Epac1‐SOCS3 pathway and IL‐6 signaling in CRF neurons on visceral hypersensitivity in rats that experienced neonatal CRD.

Suppressor of cytokine signaling 3 is a canonical anti‐inflammatory protein, with prominent immunoreactivity in the PVN and the rest of hypothalamus.^33^ Classically, SOCS3 induction occurs in response to inflammatory cues, such as IL‐6 stimulation, with subsequent activation of JAK–STAT signaling pathway.^34^ SOCS3 expression can be potentiated by macrophage‐derived IL‐6 via an endothelial‐derived Epac‐dependent pathway.^29^, ^35^ SOCS3 may inhibit IL‐6 signaling and function via gp130 or JAKs‐mediated negative feedback and exerts anti‐inflammatory effects.^36^, ^37^ IL‐6 receptor signaling includes cAMP/Epac/Rap1/SOCS3 pathway.^29^ IL‐6 enhances CRF gene expression in PVN neurons, potentiating protein secretion and HPA axis activation.^38^ In the present study, we aimed to reveal whether Epac1 modulates the SOCS3 expression in CRF neurons and the Epac1‐SOCS3 pathway in the etiology of visceral hypersensitivity.

Permanent changes for the development of visceral hypersensitivity in ELS and amplification of visceral pain responsiveness following secondary stress stimulus may be potentially involved in the development or exacerbation of IBS symptoms. Accordingly, this study utilized the neonatal‐CRD model to determine whether: (i) visceral hypersensitivity presented in male rats with a history of neonatal CRD could be exacerbated by adult secondary CRD stress, (ii) Epac1‐SOCS3 pathway affects the excitability of CRF neurons in PVN, precipitating neonatal‐CRD‐induced visceral hypersensitivity.

## 2 MATERIALS AND METHODS

### 2.1 Animals

Specific pathogen‐free (SPF) grade male and female adult Sprague–Dawley (SD) rats (220–250 g) were provided by the Experimental Animal Centre of Xuzhou Medical University. The neonatal rats were reared in a standard (42 × 25 × 23 cm) Plexiglas cage with the maternal rat until postnatal 25th day of weaning, when male rats were separated and housed (*n* = 4 per cage) with ad libitum access to food and water. However, albeit female animals could serve to mimic the female predominance of IBS in clinical scenario, the interference from estrogen and progesterone disturbances may affect the pain perception and underlying pain circuitry during the estrus cycle. Moreover, the circulating sex steroids may differentially modulate the estrogen receptor in the PVN, thus participating in the functional regulation and structural modification of PVN.^39^, ^40^ Accordingly, we only recruited male rats for experimentation. The male rats were housed in environment of a standard 12/12‐h light–dark cycle (lights on at 07:00 a.m.), controlled temperature (22°C), and humidity (50%). The animal data reporting followed the ARRIVE 2.0 guidelines (PMID:32663096). All experiments complied with the National Institutes of Health Guide for the Care and Use of Laboratory Animals (NIH Publication No. 8023, revised 1978) and the International Association for the Study of Pain to minimize the harm and the number of experimental animals and were approved by the Experimental Animal Ethics Committee at Xuzhou Medical University.

### 2.2 Animal experimental groups

The neonatal‐CRD‐induced visceral hypersensitivity has been well‐established.^14^, ^41^, ^42^ In brief, rats underwent CRD on postnatal days 8, 10, and 12 with an angioplastic balloon (20.0 mm in length; 3.0 mm in diameter) inserted via the rectum till the descending colon. The balloon was distended with 0.3 ml water at a pressure of 60 mmHg for 1 min prior to drainage and withdrawal. Distension was applied twice a day at an interval of at least 30 min. An adult CRD was applied 8 weeks later in which a 60 s/60 mmHg distension for 10 times at a 15‐s interval.^15^ Visceral hypersensitivity was assessed by balloon pressures (in mmHg) for the visceral pain threshold and abdominal withdrawal reflex (AWR) scores. Accordingly, the experiment consisted of 4 groups: neonatal‐CRD group (adult rats experiencing neonatal CRD), adult‐CRD group (rats with CRD in adulthood), dual‐CRD group (rats subjected to both neonatal and adult‐CRD stimuli), and control (naïve) group.

### 2.3 Determination of abdominal withdrawal reflex (AWR) and visceral pain threshold in adulthood

Rats were fasted for 12 h with water ad libitum before assessment. A homemade latex balloon (2 cm in length and 2 cm in diameter) fastened to the catheter was connected to a sphygmomanometer and a syringe via a Y‐shaped joint. The balloon was inserted through the rectum till the descending colon prior to distension and withdrawal. The isoflurane (3%–5%) was applied to induce anesthesia during the implantation of the balloon, and CRD was induced while rats were fully awake. A visceromotor response (AWR score) to graded CRD stimuli (10–50 mmHg, 20 s each) was assessed at an interval of 4 min (0, representing immobility; 1, only slight head movement; 2, abdominal contraction with contact with the table; 3, evident abdominal contraction and elevation; and 4, dorsal arching and/or the pelvic elevation.^14^ For the time course after administration, the interval was reduced to 1–2 min to ensure the accuracy of concentration–time curve as soon as possible. The pain threshold was defined as a pressure to elicit visible contraction of the abdominal wall, that is, AWR score of 3. The measurement was conducted in triplicate, with the average score calculated for further analysis.

### 2.4 Reagents

The profiles of reagents and antibodies (Tables 1 and 2).

**TABLE 1:** Drugs for intra‐PVN microinjection

Drugs	Concentration	Volume (unilateral)	Company, Cat. No.
ESI‐09	1.0 μg/μl	0.25 μl	TOCRIS, 4773
HJC‐0350	1.0 μg/μl	0.25 μl	TOCRIS, 4844
8‐pCPT	1.0 μg/μl	0.25 μl	TOCRIS, 4853
IL‐6	200 μg/μl	0.3 μl	Abcam, ab9324
IL‐6 NA	10 ng/μl	0.3 μl	Abcam, ab11449
Epac1‐siRNA	0.5 μg/μl	3 μl	GenePharma Co. Ltd.
SOCS3‐siRNA	0.5 μg/μl	3 μl	GenePharma Co. Ltd.
AAV‐SOCS3	1.17 E + 12 vg/ml	0.5 μl	Genechem Co. Ltd.
rAAV‐CRF	2.04 E + 12 vg/ml	0.5 μl	Shumi Brain Science and Technology Co. Ltd.

**TABLE 2:** Antibodies for Immuno‐staining and Western blotting

Antigen	Host	Dilution	Company, Cat. No.
Epac1	Rabbit, polyclonal	1:1000 (WB) 1:200 (IF)	Abcam, ab21236
Epac2	Rabbit, polyclonal	1:1000 (WB)	Abcam, ab21238
Rap1	Rabbit, monoclonal	1:1000 (WB)	Abcam, ab187659
SOCS3	Rabbit, polyclonal	1:1000 (WB)	Abcam, ab 16030
	Mouse, monoclonal	1:200 (IF)	Abcam, ab14939
CRF	Rabbit, polyclonal	1:200 (IF)	Abcam, ab8901
	Mouse, monoclonal	1:1000 (WB)	Abcam, ab35748
IL‐6	Mouse, monoclonal	1:1000 (WB)	Abcam, ab9324
c‐Fos	Rabbit, monoclonal	1:1000 (IF)	CST, #2250
	Mouse, monoclonal	1:1000 (IF)	NOVUS, NBP2‐50037
GFAP	Rabbit, polyclone	1:200	Abcam, ab7260
Iba‐1	Rabbit, monoclonal	1:200	Abcam, ab178847
β‐Actin	Rabbit, polyclone	1:1000 (WB)	Biosharp, BL0005A
GAPDH	Mouse	1:1000 (WB)	ABclonal Co., AC001
AlexaFluor® 488 IgG	Mouse	1:400 (IF)	Life
AlexaFluor® 594 IgG	Rabbit	1:400 (IF)	Life
Horseradish eroxidase	Rabbit	1:1000 (WB)	Beyotime, A0208
IgG (H + L)	Mouse	1:1000 (WB)	Beyotime, A0216
Alkaline phosphatase	Rabbit	1:1000 (WB)	Zhongshan Jinqiao, ZB‐2308
	Mouse	1:1000 (WB)	ZB‐2310

### 2.5 Intra‐PVN microinfusion

Under isoflurane (3%–5%) anesthesia, each rat was secured with the head in horizontal position on a stereotaxic apparatus (RWD). Drugs were bilaterally microinfused into PVN (A/P: −1.5 mm, L/R: ± 0.4 mm, D/V: −7.8 mm from bregma) (Paxinos and Watson, 2007) in 3 min (velocity = 0.167 μl/min). The needle was retained in site for 10 min to prevent drug reflux prior to skin closure. The site per injection of PVN was verified histologically for each brain, and rats with incorrect cannulation were excluded from data analysis. The overall success rate of bilateral PVN injections was 81.8% (Figure S1).

### 2.6 Recording of PVN CRF neuronal firing

Corticotropin‐releasing factor neuronal firing was recorded in PVN slices via cell‐attached electrophysiology. In brief, 21 days after microinjection of CRF‐specific promoter virus (rAAV‐CRF‐EYFP‐WPRE‐PA) in PVN, rats were deeply anesthetized followed by decapitation and brain isolation. PVN slices at 300 μm were collected with a vibrating microslicer (VT1000S, Leica, Germany) in cold cutting solution (mM: 80 NaCl, 3.5 KCl, 4.5 MgSO_4_, 0.5 CaCl_2_, 1.25 NaH_2_PO_4,_ 90 sucrose, 25 NaHCO_3_, 10 glucose) and incubated for 15 min at 32°C and then incubated in artificial cerebrospinal fluid (aCSF [mM]: 126 NaCl, 2.5 KCl, 1.2 NaH_2_PO_4_, 1.2 MgSO_4_, 26 NaHCO_3_, 10 glucose and 2.4 CaCl_2_ and bubbled with 95% O_2_ and 5% CO_2_) at 35°C for at least 1 h prior to transfer into a recording chamber perfused with aCSF (at 32–33°C, flow velocity = 2.5 ml/min).

Patch pipettes (3–5 mV) for cell‐attached recordings were filled with an internal solution containing: (in mM) 115 K‐gluconate, 10 phosphocreatine, 10 HEPES, 1.5 MgCl_2_, 20 KCl, 2 Mg‐ATP, and 0.5 GTP. PVN CRF neurons were identified by the fluorescence of AAV and electrophysiological characters.^43^ Signals were band‐pass filtered at 300 Hz‐1 kHz and Bessel filtered at 10 kHz with a Multiclamp700B amplifier. Data acquisition and analysis were conducted by Clampex and Clampfit 10 (Axon Instruments).

### 2.7 Plasma hormone measurements

Venous blood samples were collected from the right atrium from 12:00 to 16:00 and transferred into EP tubes containing EDTA and then stored at −80°C until shipment for radioimmunoassay. Plasma CRF, ACTH, and corticosterone (CORT) were determined by radioimmunoassay at Huaying Biotechnology Research Institute (Beijing, China) following assay kit instruction: CRF (HY‐10175), ACTH (HY‐10057), and CORT (HY‐114).

### 2.8 Immunofluorescent staining

Under deep anesthesia, rats were transcardially perfused with 0.9% saline (100 ml/100 g), followed by 4% paraformaldehyde in phosphate buffer (100 ml/100 g). Epac1, SOCS3, and CRF are mainly located in the cytoplasm. Brain tissue was equilibrated in 30% sucrose solution for 5–7 days after fixation in 4% paraformaldehyde phosphate buffer for 24 h at 4°C. Brain was sliced at 30‐μm thickness with a cryostat (Leica CM1800; Heidelberg, Germany). There was a margin of 150 μm between the sections to preclude duplicate calculation. Selected slices were rinsed twice with PBS for 5 min at room temperature (r/t) and permeabilized with TBS for 5 min and then incubated with 200 μl 10% donkey serum for 2 h before incubation with the primary antibody at 4°C for 24 h (Table 2). Secondary antibodies were added to the corresponding sections in a dark chamber and incubated at r/t for 2 h. DAPI (4,6‐diamino‐2‐phenylindole) was added for DNA counterstaining before cover slipping. Tissue sections from each group were visualized with a confocal laser microscope (FV1000, Olympus). We performed randomized double‐blind experiments and adopted a reliable and reproducible method to avoid research bias in the study.

### 2.9 Western blot analysis

Western blot analysis was adopted to characterize the protein expression in PVN and pituitary. The PVN is located dorsal to the supraoptic nucleus, adjacent to the third ventricle. The pituitary gland is an ovoid body located in the ventral hypothalamus. Brain region containing PVN or pituitary was harvested and lysed with synaptic protein extract (1 ml/100 mg) containing PMSF. Homogenates were centrifuged at 12,000 *g* for 15 min at 4°C, and supernatant was collected. 30 μg protein lysis was separated by SDS‐PAGE gels before transference onto a PVDF membrane. After blockade with 5% non‐fat milk powder (VICMED) for 2 h, membrane was incubated with primary antibodies for 24 h at 4°C. After rinses with washing buffer, membrane was incubated with HRP‐conjugated secondary antibodies (1: 2000) or AP‐conjugated secondary antibody (1: 1000) for 2 h for 40 min. The protein bands were visualized by a chemiluminescence kit analyzed by ProPlus image analysis system (NIH, Bethesda, MD, US).

### 2.10 Enzyme‐linked immunosorbent assay (ELISA)

The expression level of IL‐6 was quantified by ELISA. Briefly, the PVN was collected and homogenized in a RIPA lysis buffer (P0013B, Beyotime, China). The homogenate was centrifuged at 12,000 *g* (4°C) for 15 min. The supernatant was collected and assayed with IL‐6 assay kits (MyBioSource) as per the manufacturer's instructions. Cytokine concentration is expressed as pg/mg protein.

### 2.11 Quantitative real‐time polymerase chain reaction (qRT‐PCR)

The mRNA levels in PVN were determined by qRT‐PCR. Epac1, rap1, SOCS3, CRF, and glyceraldehyde‐3‐phosphate dehydrogenase (GAPDH) mRNAs were purified by RNA extraction and purification kit (Sangon Biotech) and reversed‐transcribed using M‐MLV Reverse Transcriptase (Thermo Fisher Scientific) and dT primers. The RNA purity was detected by the ratio of the absorbance at 260 nm and 280 nm (1.8 < 260/280 ratio <2.1). A template of 10 pg to 100 ng of total RNA was reversely transcribed into cDNA with Hiscript RTSuperMix for qPCR. The cDNA results were standardized to GAPDH measured on the same sample. PCR amplification was performed with Taq polymerase using 40 cycles at 95°C for 10 s, 60°C for 30 s, and 72°C for 30 s. The PCR primers (Invitrogen Biotech Co. Ltd.) were listed in Table 3:

**TABLE 3:** Primers used in quantitative PCR

Name	Sequence
Epac1‐sense	5′‐ GAGACTGGAAGAACACGGCA −3’
Epac1‐antisense	5’‐ TCCGGTCTCATAGCCTCCAA −3’
Rap1‐sense	5’‐ CTTGGTTCAGGAGGCGTGG −3’
Rap1‐antisense	5’‐ AGCATGCACTGTTGGCAATC ‐3′
SOCS3‐sense	5′‐ CTTTACCACCGACGGAACCT −3′
SOCS3‐antisense	5′‐ CCGTTGACAGTCTTCCGACA −3′
CRF‐sense	5′‐ GCAGCGGGACTTCTGTTGA −3′
CRF‐antisense	5′‐ CGCAGCCGTTGAATTTCTTG −3′
GAPDH‐sense	5′‐ TGAAGCAGGCATCTGAGG −3′
GAPDH‐antisense	5′‐ CGAAGGTGGAAGAGTGGGAG −3’

The qRT‐PCR was performed with real‐time SYBR Green PCR technology using LightCycler® 480II (Roche). The reaction mixtures contained diluted cDNA, SYBR® Premix Ex TaqII (TliRNaseH Plus) (2×), 10 μM of each gene‐specific primer, and nuclease‐free water in a final volume of 10 μl.

### 2.12 Statistical analysis

Data are expressed as mean ± SD. Kolmogorov–Smirnov (K‐S) tests were used for the assessment of normality and data distribution. All of the following statistical tests were assumed to be a normal distribution. Molecular expression levels were analyzed by one‐way ANOVA. The time courses of pain thresholds were analyzed with a two‐way repeated measures ANOVA, followed by post hoc Bonferroni multiple comparisons in case of significance. Student's *t*‐test was used to calculate group difference. All statistical tests were conducted with SPSS 16.0 (IBM) software package. *p* < 0.05 was considered statistically significant.

## 3 RESULTS

### 3.1 Predisposition of neonatal and/or adult CRD to the development of visceral hypersensitivity

**FIGURE 1:**
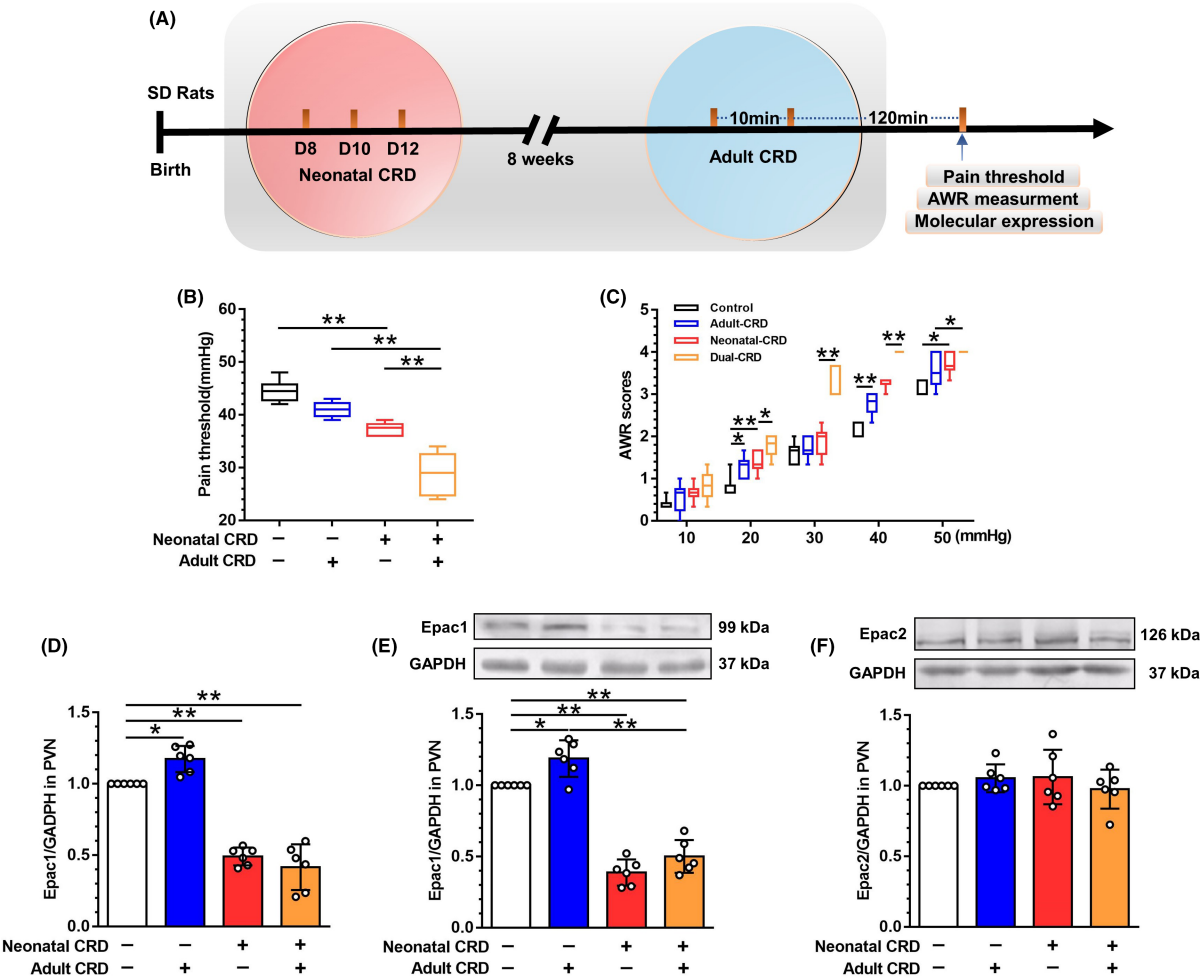
Downregulated Epac1‐mediated neonatal‐CRD‐induced visceral hypersensitivity. (A) Schematic description of time course of experiments. Neonatal CRD was performed on postnatal days 8, 10, and 12. Adult CRD was applied between weeks 8 and 10. At 120 min after adult CRD, all rats were sacrificed with brain samples isolated for RT‐PCR, Western blotting, ELISA, and immunofluorescent staining. (B) Neonatal‐ and dual‐CRD rats presented a significant decrease in pain threshold vs. control and adult‐CRD rats, respectively (*n* = 8). (C) Neonatal‐ and dual‐CRD rats displayed a significant increase in AWR score (*n* = 8). (D) Epac1 mRNA expression was significantly decreased in neonatal‐CRD and dual‐CRD groups vs. control group (*n* = 6). (E) Epac1 protein expression was significantly decreased in neonatal‐CRD and dual‐CRD groups vs. control group (*n* = 6). (F) Epac2 protein had no significant alterations in each group (*n* = 6). Data are presented as the mean ± SD. *p < 0.05, **p < 0.01 vs. indicated group

We employed CRD to establish rat model of ELS‐induced visceral hypersensitivity in neonatal SD rats on postnatal days 8, 10, and 12. On maturity (8th–10th week), secondary CRD was performed in rats having undergone neonatal CRD. Visceral hypersensitivity was quantified 120 min after secondary CRD via balloon pressures (in mmHg) for the pain thresholds and abdominal withdrawal reflex (AWR) scores (Figure 1A). Indeed, the naïve adult rats subjected to CRD exhibited visceral hypersensitivity, which was rescued at 120 min thereafter, whereas the visceral hypersensitivity in adult rats experiencing neonatal CRD was not reversed and was exacerbated by secondary CRD stimulation. Rats experiencing dual‐CRD presented a significant decrease in visceral pain threshold (F [3,20] = 46.93, *p* < 0.01; Figure 1B) as well as the elevated AWR scores (Figure 1C), compared with naïve rats or rats that experienced either neonatal or adult‐CRD rats.

For the assessment of Epac expression in PVN, naïve and neonatal‐CRD rats underwent adult CRD (hence adult‐CRD and dual‐CRD groups) and were sacrificed 120 min thereafter, with brain samples collected, in parallel with maneuver of brain isolation in control and neonatal‐CRD groups. One‐way ANOVA showed a significant difference in Epac1 mRNA level (F [3,20] = 88.2, *p* < 0.01, Figure 1D). Post hoc Bonferroni multiple comparisons showed that neonatal‐CRD and dual‐CRD group showed a significant decrease in Epac1 mRNA expression (*p* = 0.01). The adult‐CRD group showed a significant increase in Epac1 mRNA expression (*p* = 0.04). Consistently, a one‐way ANOVA showed a significant difference in Epac1 protein levels (F [3,20] = 93.64, *p* < 0.01; Figure 1E). Post hoc Bonferroni multiple comparisons showed that rats experienced neonatal CRD or dual CRD presented a significant decrease in Epac1 protein expression. The adult‐CRD group showed a significant increase in Epac1 protein expression (*p* = 0.02). No between‐group difference was observed in Epac2 protein expression (F [3,20] = 0.62, *p* > 0.99; Figure 1F). These results suggested that Epac1 in PVN neurons could modulate neonatal‐CRD‐induced visceral hypersensitivity.

### 3.2 Activation of Epac1 in PVN alleviated the visceral hypersensitivity and decreased the excitability of PVN CRF neurons in neonatal‐CRD rats

**FIGURE 2:**
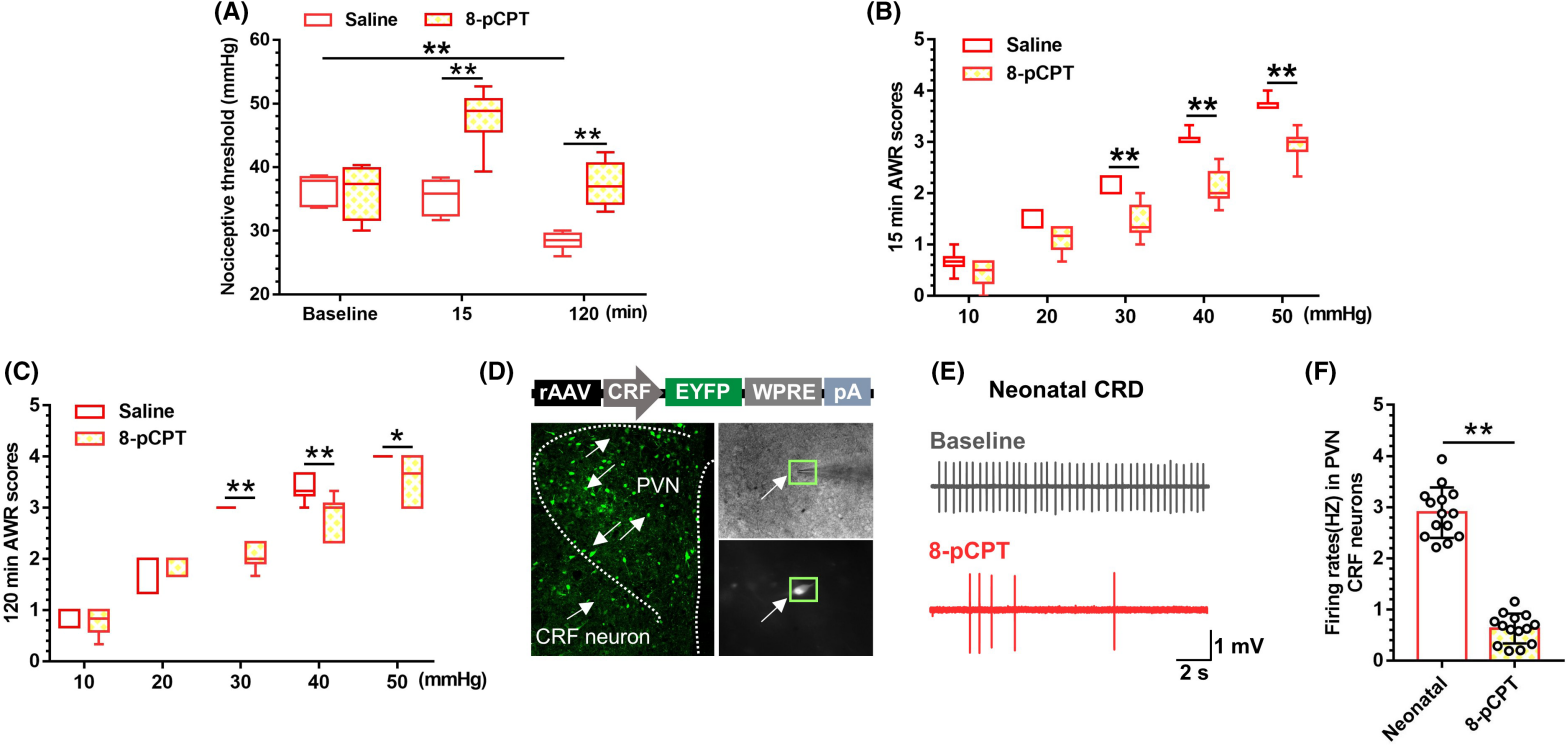
Activation of Epac1 in PVN alleviated the visceral hypersensitivity and decreased the excitability of PVN CRF neurons in neonatal‐CRD rats. (A) Epac agonist 8‐pCPT significantly elevated the visceral nociceptive threshold 15 min after administration and 120 min after adult‐CRD stimuli in neonatal‐CRD group (*n* = 6). (B) Epac agonist 8‐pCPT significantly decreased the AWR scores at 30, 40, and 50 mmHg 15 min after administration (*n* = 6). (C) Epac agonist 8‐pCPT significantly decreased the AWR scores at 30, 40, and 50 mmHg 120 min after adult‐CRD stimuli in neonatal‐CRD group (*n* = 6). (D) Electrophysiological recording of specific AAV labeled CRF neurons. (E) Schematic diagram of discharge of CRF neurons before and after 8‐pCPT administration. (F) In vitro electrophysiology revealed that 8‐pCPT perfusion inhibited the firing of CRF neurons in PVN from neonatal‐CRD group (*n* = 14 cells from 6 rats). Data are presented as the mean ± SD. *p < 0.05, **p < 0.01 vs. indicated group

To evaluate the role of Epac1 in visceral hypersensitivity, we employed pharmacological intervention to reversibly modulate Epac protein function with Epac agonist cAMP analog 8‐pCPT in neonatal‐CRD rats (Figure 2A). A two‐way repeated measures ANOVA showed a significant difference in time (F [2,20] = 26.38, *p* < 0.01), treatment (F [1,10] = 29.24, *p* < 0.01) and time × treatment interaction (F [2,20] = 15.63, *p* < 0.01). Post hoc Bonferroni multiple comparisons showed a significant decrease in pain threshold 120 min after vehicle treatment in neonatal‐CRD rats (*p* < 0.01), in contrast to the increase in pain threshold 15 min after intra‐PVN infusion of 8‐pCPT (*p* < 0.01). Secondary adult CRD was undertaken at 15 min after 8‐pCPT administration. At 120 min thereafter, the pain threshold was restored in 8‐pCPT group compared with vehicle group (*p* < 0.01). Accordingly, the AWR scores were remarkably decreased in neonatal‐CRD rats after 8‐pCPT treatment (Figure 2B,C). 15 min after drug administration, a two‐way repeated measures ANOVA showed a significant difference in time (F [4,40] = 278.7, *p* < 0.01), treatment (F [1,10] = 46.05, *p* < 0.01) and time × treatment interaction (F [4,40] = 5.42, *p* < 0.01). Post hoc Bonferroni multiple comparisons showed a significant decrease in AWR scores at 30, 40, and 50 mmHg 15 min after 8‐pCPT treatment in neonatal‐CRD rats (*p* < 0.01). Likewise, 120 min after drug administration, a two‐way repeated measures ANOVA showed a significant difference in time (F [4,40] = 239.8, *p* < 0.01), treatment (F [1,10] = 18.84, *p* < 0.01) and time × treatment interaction (F [4,40] = 8.24, *p* < 0.01). Post hoc Bonferroni multiple comparisons showed a significant decrease in AWR scores at 30, 40 (*p* < 0.01) and 50 mmHg (*p* = 0.03) 120 min after 8‐pCPT treatment in neonatal‐CRD rats. Subsequently, we assessed the electrophysiological properties of CRF neurons in PVN. The CRF neurons in brain slices were labeled by a CRF‐specific promoter virus (Figure 2D). As illustrated in Figure 2E,F 8‐pCPT reduced the firing frequency of PVN CRF neurons in neonatal‐CRD group (t [26] = 14.83, *p* < 0.01). These findings demonstrated that the activation of Epac decreased the excitability of PVN CRF neurons, abated visceral hypersensitivity, and prevented pain precipitation via 8‐pCPT treatment.

### 3.3 Inhibition of Epac1 in PVN contributed to visceral hypersensitivity and increased the excitability of PVN CRF neurons

**FIGURE 3:**
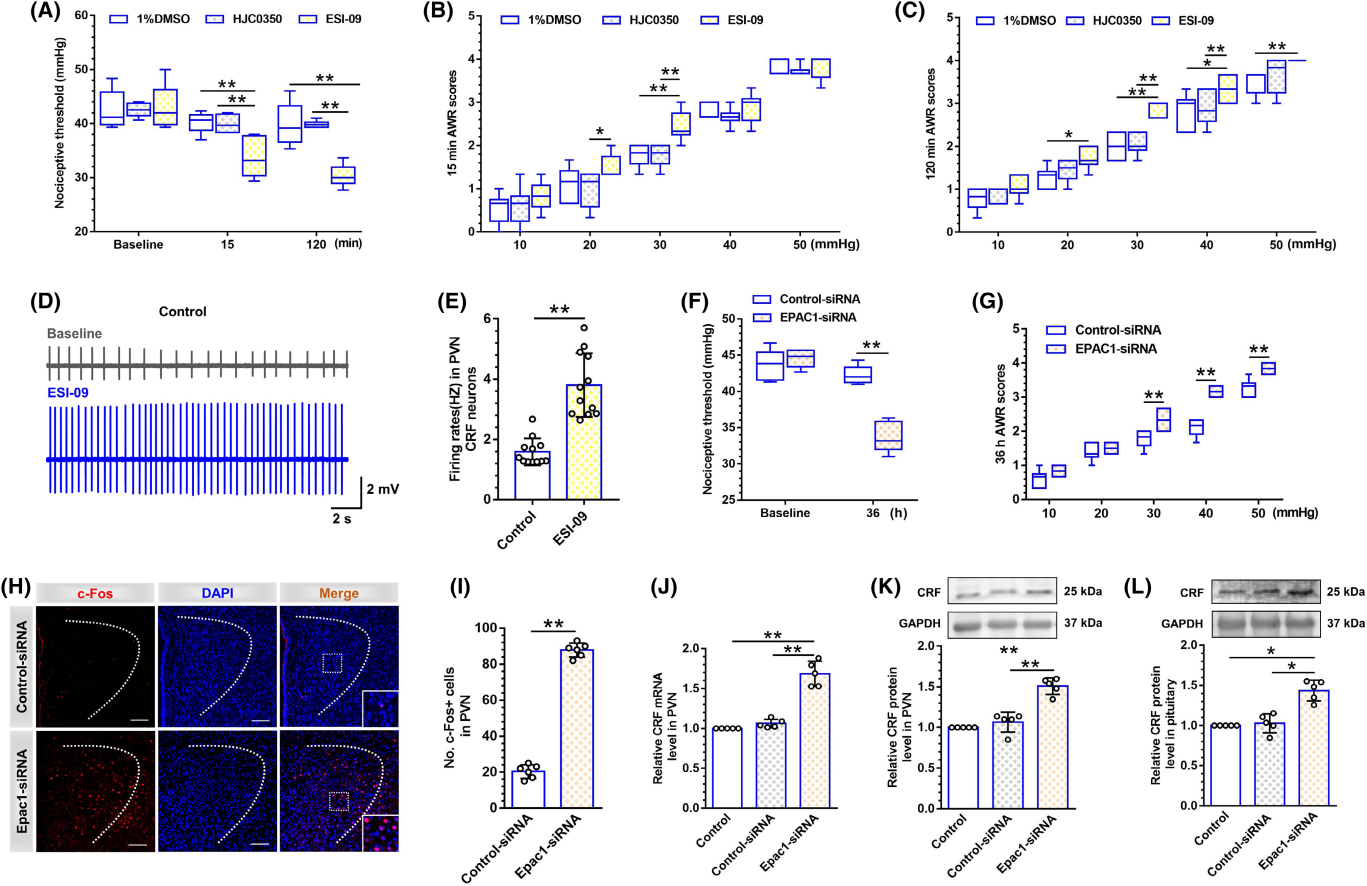
Inhibition of Epac1 in PVN facilitated visceral hypersensitivity and increased the excitability of PVN CRF neurons in control rats. (A) Epac antagonist ESI‐09 reduced the nociceptive threshold 15 min after administration and 120 min after adult‐CRD stimuli in control group; however, Epac2 antagonist HJC0350 had no evident effect on the pain threshold in control group (*n* = 6). (B) ESI‐09 significantly elevated the AWR scores at 20 and 30 mmHg 15 min after administration in control group (*n* = 6). (C) ESI‐09 significantly elevated the AWR scores at 20, 30, 40, and 50 mmHg 120 min after adult‐CRD stimuli in control group (*n* = 6). (D) Schematic diagram of discharge of CRF neurons before and after ESI‐09 administration. (E) In vitro electrophysiology revealed that ESI‐09 perfusion increased the firing of CRF neurons in PVN from control group (*n* = 12 cells from 6 rats). (F) The nociceptive threshold significantly decreased by 36 h after Epac1‐siRNA injection into PVN in naïve rats (*n* = 6). (G) The AWR scores were significantly elevated at 30, 40, and 50 mmHg 36 h after Epac1‐siRNA injection into PVN in naïve rats (*n* = 6). (H) Immunofluorescent analyses showed the activated PVN neurons were labeled with c‐Fos 36 h after Epac1‐siRNA injection in naïve rats (*n* = 3–4 sections from 5 rats, scale bar = 100 μm). (I) Cell counts of c‐Fos‐positive neurons in PVN 36 h after Epac1‐siRNA injection (*n* = 6). (J) CRF mRNA in PVN from control group was significantly increased 36 h after Epac1‐siRNA, as well as (K) CRF protein expression. Meanwhile, (L) pituitary CRF protein was significantly elevated (*n* = 5). Data are presented as the mean ± SD. *p < 0.05, **p < 0.01 vs. indicated group

Next, we adopted pharmacological intervention via Epac antagonists ESI‐09 and HJC0350 to explore the effects of Epac in control rats (Figure 3A). Two‐way repeated measures ANOVA showed significant differences in time (F [2,30] = 45.29, *p* < 0.01), treatment (F [2,15] = 10.75, *p* < 0.01) and time × treatment interaction (F [4,30] = 13.42, *p* < 0.01). Post hoc Bonferroni multiple comparisons showed a similar reduction of the nociceptive threshold at 15‐min post‐administration of ESI‐09 in control rats, with no sign of recovery even at 120 min after CRD (*p* < 0.01), whereas the Epac2 antagonist HJC0350 failed to alter visceral nociception (*p* > 0.05). Accordingly, the AWR scores were elevated in normal rats after ESI‐09 treatment (Figure 3B). 15 min after drug administration, two‐way repeated measures ANOVA showed significant differences in time (F [4,60] = 370.6, *p* < 0.01), treatment (F [2,15] = 5.79, *p* = 0.01) and time × treatment interaction (F [8,60] = 1.58, *p* = 0.15). Post hoc Bonferroni multiple comparisons showed a significant increase in AWR scores at 20 (*p* = 0.02) and 30 mmHg 15 min after ESI‐09 treatment in normal rats (*p* < 0.01). At 120 min after drug administration, two‐way repeated measures ANOVA showed significant differences in time (F [4,60] = 387.5, *p* < 0.01), treatment (F [2,15] = 14.33, *p* < 0.01) and time × treatment interaction (F [8,60] = 1.81, *p* = 0.10, Figure 3C). Post hoc Bonferroni multiple comparisons showed a significant increase in AWR scores at 20 (*p* = 0.03), 30 (*p* < 0.01), 40 (1% DMSO vs. ESI‐09, *p* < 0.01; HJC0350 vs. ESI‐09, *p* = 0.03) and 50 (*p* < 0.01) mmHg 120 min after ESI‐09 treatment in neonatal‐CRD rats. Consistently, the electrophysiological results demonstrated that ESI‐09 perfusion elevated the firing frequency of PVN CRF neurons in the control group (t [22] = 6.69, *p* < 0.01, Figure 3D,E).

Next, Epac1‐siRNA was intra‐PVN injected in control rats and Epac1 knockdown was verified by the decreased Epac1 protein expression (t [10] = 14.76, *p* < 0.01; Figure S2). In naïve rats, genetic knockdown Epac1 protein expression with siRNA reduced nociceptive threshold (Figure 3F). Similarly, the AWR scores were elevated in normal rats after Epac1‐siRNA treatment (Figure 3G). c‐Fos protein and DAPI were double stained and visualized by immunofluorescence (Figure 3H). The density of c‐Fos‐positive cells in PVN was significantly increased (t [10] = 30.58, *p* < 0.01; Figure 3I). Furthermore, 36 h after Epac1‐siRNA microinjection into PVN, the expression profiles of CRF mRNA and protein were examined. One‐way ANOVA showed a significant difference (PVN CRF mRNA: F (2,12) = 77.71, *p* < 0.01, *n* = 5; PVN CRF protein: F (2,12) = 45.96, *p* < 0.01, *n* = 5; pituitary CRF protein: F (2,12) = 29.23, *p* < 0.01, *n* = 5; Figure 3J–L). Post hoc Bonferroni multiple comparisons showed that rats receiving Epac1‐siRNA microinjection presented a significant increase in the expression of PVN CRF mRNA, PVN CRF protein, and pituitary CRF protein, as compared to the control rats. These results suggested that Epac1 in PVN CRF neurons could modulate the CRF levels and activity of HPA axis.

### 3.4 The implication of SOCS3 underexpression in visceral hypersensitivity in neonatal‐CRD rats

**FIGURE 4:**
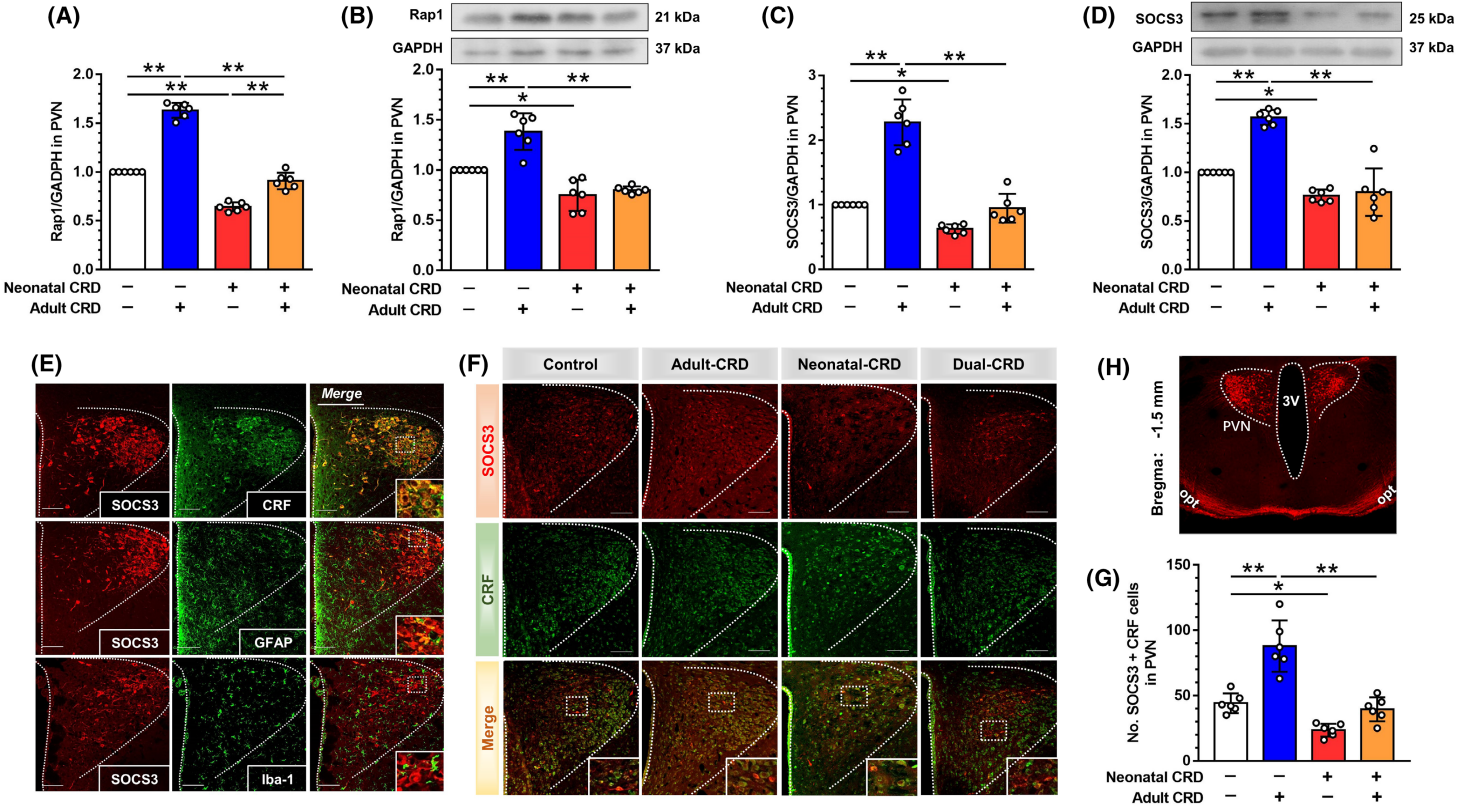
Downregulation of downstream effector SOCS3 participated in visceral hypersensitivity in neonatal‐CRD or dual‐CRD rats. (A–D) The expression of Rap1 mRNA, Rap1 protein, SOCS3 mRNA, and SOCS3 protein in PVN in each group (*n* = 6). (E) SOCS3 double‐labeled with CRF and GFAP, rather than Iba‐1 (Scale bar = 100 μm). (F) Co‐expression of CRF with SOCS3 in PVN in each group. (G) Statistical diagram for SOCS3 and CRF co‐labeling (*n* = 6, scale bar = 100 μm). (H) Schema of PVN, and immunofluorescent staining of SOCS3 in PVN. Data are presented as the mean ± SD. *p < 0.05, **p < 0.01 vs. indicated group

We explored whether Epac1 participated in neonatal‐CRD‐induced hypersensitivity via the SOCS3 pathway. SOCS3 protein expression was upregulated 120 min after 8‐pCPT administration in neonatal‐CRD rats (t [10] = 7.17, *p* < 0.01; Figure S3). In contrast, SOCS3 was downregulated 120 min after ESI‐09 injection in control rats (t [10] = 19.03, *p* < 0.01; Figure S4). These findings implied that SOCS3 might be involved in the neonatal‐CRD‐induced visceral hypersensitivity. Accordingly, we assessed the expression of downstream Rap1‐SOCS3 pathway among groups. The mRNA of Rap1 increased in the adult‐CRD rats (*p* < 0.01, Figure 4A) and decreased in neonatal‐CRD rats (*p* < 0.01) compared with the control rats. Moreover, a significant increase in Rap1 mRNA expression was observed in adult‐CRD rats compared with dual‐CRD rats (*p* < 0.01). In parallel, the protein of Rap1 increased in the adult‐CRD rats (*p* < 0.01, Figure 4B) and decreased in neonatal‐CRD rats (*p* < 0.05) compared with the control rats. Moreover, adult‐CRD rats presented a significant increase in Rap1 protein expression compared with dual‐CRD rats (*p* < 0.01). The mRNA of SOCS3 increased in the adult‐CRD rats (*p* < 0.01, Figure 4C) and decreased in neonatal‐CRD rats (*p* < 0.01) compared with the control rats. SOCS3 protein expression significantly decreased in neonatal‐CRD rats compared with the control group (*p* = 0.03, Figure 4D), but remarkably increased in adult‐CRD rats as compared to the control as well as the dual‐CRD groups (*p* < 0.01). To determine the SOCS3 distribution and co‐localization with relevant markers in PVN, the expression profiles of SOCS3, GFAP, Iba‐1, and CRF neurons in PVN slices were visualized. SOCS3 was sufficiently co‐expressed with CRF neurons, modestly with GFAP, and scarcely with Iba‐1 in adult‐CRD rats (Figure 4E). SOCS3 and CRF were double‐labeled by immunofluorescence (Figure 4F), which revealed a significant difference in SOCS3 protein (F [3,20] = 32.82, *p* < 0.01, Figure 4G). Post hoc Bonferroni multiple comparisons showed that SOCS3 protein co‐labeled with CRF was significantly decreased in PVN in neonatal‐CRD group (*p* = 0.04), but was increased in adult‐CRD group (*p* < 0.01). Meanwhile, the mapping of PVN was delineated in a coronal plane and PVN contour was visualized by immunofluorescence of SOCS3 (Figure 4H). Most importantly, the median eminence received amounts of axonal projections mainly derived from CRF‐containing cell somata in PVN, implying that PVN SOCS3‐positive CRF neurons acted at the pituitary to regulate ACTH secretion into the circulation, resulting in the activation of HPA axis. All these findings demonstrated that SOCS3 was expressed in CRF neurons in PVN, with the downregulation of SOCS3 expression in CRF neurons in PVN implicated in the neonatal‐CRD‐induced chronic visceral hypersensitivity.

### 3.5 Activation of SOCS3 in PVN contributed to the alleviated visceral hypersensitivity and the decreased excitability of PVN CRF neurons in neonatal‐CRD rats

**FIGURE 5:**
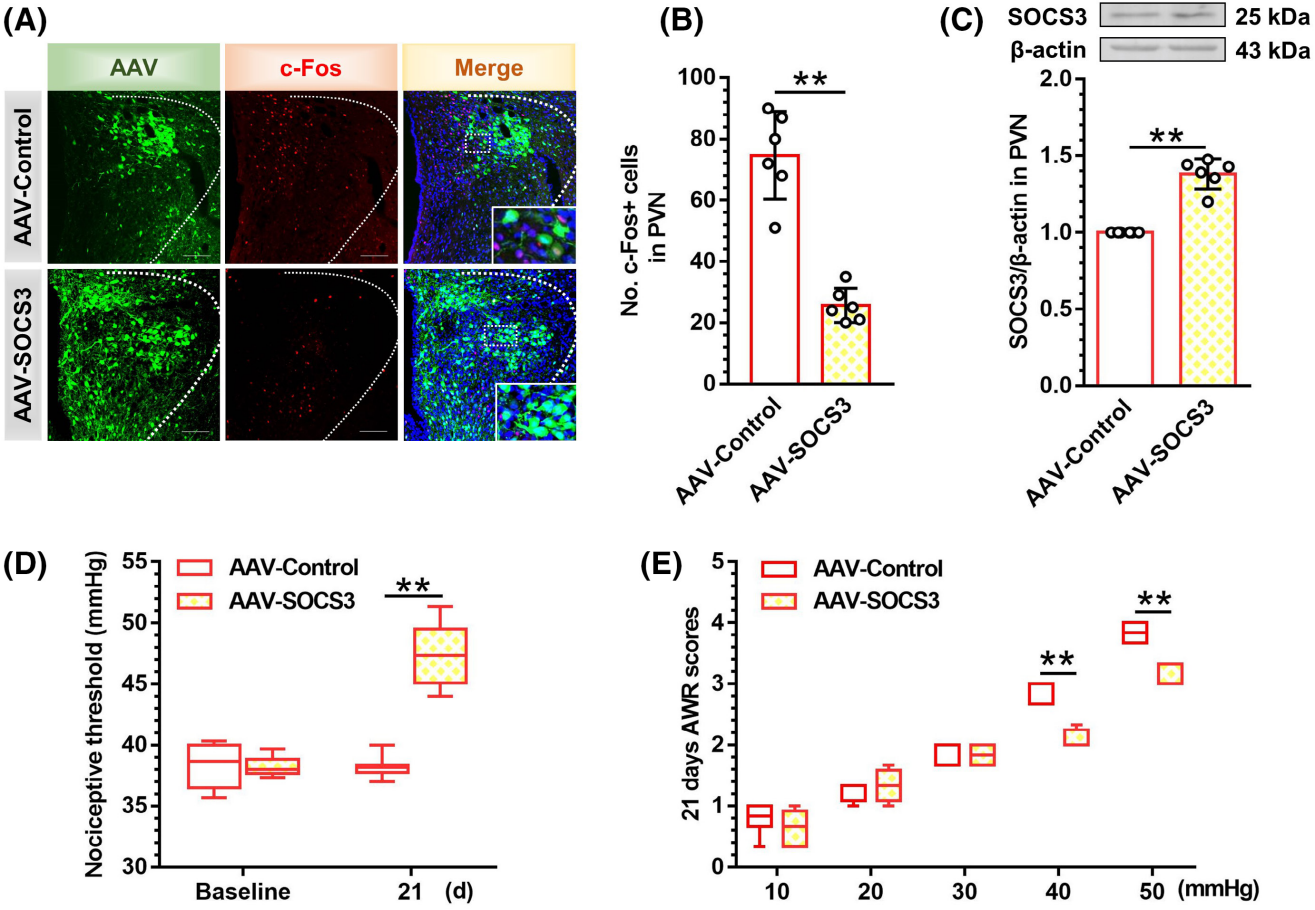
Activation of SOCS3 in PVN alleviated visceral hypersensitivity with the decreased excitability of PVN CRF neurons in neonatal‐CRD rats. (A) Typical immunofluorescent image of c‐Fos protein after AAV9‐SOCS3 intervention (Scale bar = 100 μm). (B) c‐Fos was significantly decreased in PVN in neonatal‐CRD group 21 days after AAV‐SOCS3 injection (*n* = 6). (C) Overexpressed SOCS3 protein was detected in PVN 21 days after AAV‐SOCS3 injection in neonatal‐CRD group (*n* = 6). (D) The nociceptive threshold elevated 21 days after AAV‐SOCS3 injection in neonatal‐CRD group (*n* = 6). (E) The AWR scores were decreased at 40 and 50 mmHg 21 days after AAV‐SOCS3 injection. Data are presented as the mean ± SD. **p < 0.01 compared with indicated group

To clarify the implications of SOCS3 expression in PVN, we microinjected AAV‐SOCS3 into PVN to overexpress SOCS3 in neonatal‐CRD group. Immunofluorescence triplot of SOCS3‐, c‐Fos‐, and DAPI‐labeled neurons was visualized 21 days after AAV‐SOCS3 treatment (Figure 5A). Rats undergoing AAV‐SOCS3 administration presented a significant decrease in c‐Fos protein expression in PVN as compared to the AAV‐control group (t [10] = 7.80, *p* < 0.01; Figure 5B). To verify the functional expression of SOCS3, independent samples Student's *t*‐test showed that SOCS3 protein was significantly upregulated 21 days after AAV‐SOCS3 injection (t [10] = 9.52, *p* < 0.01; Figure 5C). In line with our hypothesis, the nociceptive threshold was elevated at 21‐day post‐injection of AAV‐SOCS3 in neonatal‐CRD rats (*p* < 0.01, Figure 5D). The AWR scores at 40 and 50 mmHg were decreased 21 days after AAV‐SOCS3 treatment in neonatal‐CRD rats (Figure 5E). In summary, neonatal CRD could elicit long‐lasting visceral hypersensitivity via the downregulation of SOCS3, whereas interventions with intra‐PVN injection of exogenous AAV‐SOCS3 reversed this hypersensitivity.

### 3.6 Inhibition of SOCS3 in PVN contributed to visceral hypersensitivity and the increased excitability of PVN neurons in naïve rats

**FIGURE 6:**
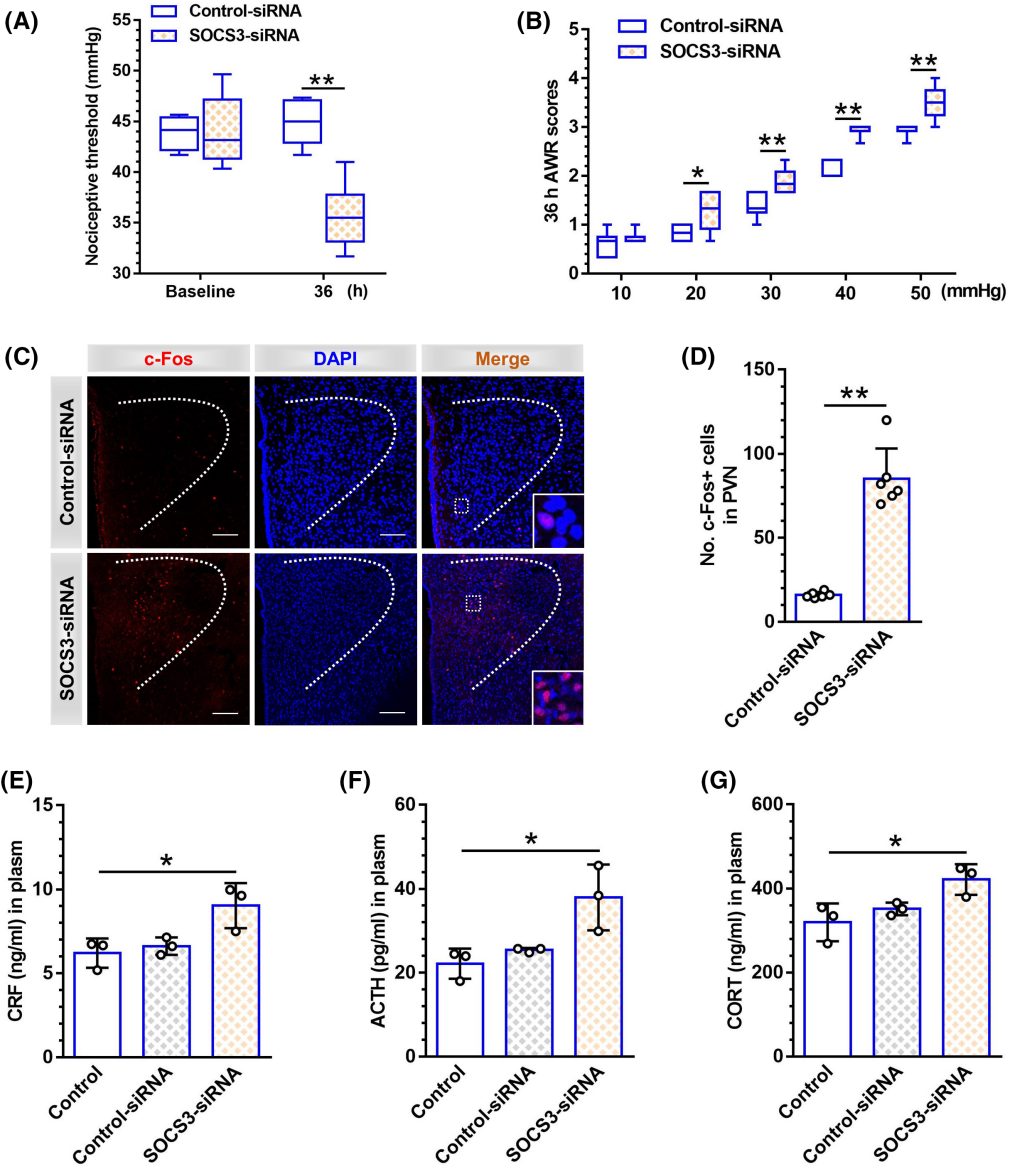
Inhibition of SOCS3 in PVN facilitates visceral hypersensitivity with the increased excitability of PVN neurons in control rats. (A) The nociceptive threshold declined 36 h after SOCS3‐siRNA injection in naïve rats (*n* = 6). (B) The AWR scores were elevated at 20, 30, 40, and 50 mmHg 36 h after SOCS3‐siRNA injection in naïve rats (*n* = 6). (C) Immunofluorescence of c‐Fos and DAPI in PVN 36 h after SOCS3‐siRNA injection in naïve rats (*n* = 3–4 sections from 6 rats, scale bar = 100 μm). (D) Cell counts of c‐Fos‐positive neurons in PVN 36 h after SOCS3‐siRNA injection (*n* = 6). (E–G) CRF, ACTH, and CORT in peripheral plasma were significantly increased 36 h after intra‐PVN injection of SOCS3‐siRNA (*n* = 3). Data are presented as the mean ± SD. *p < 0.05, **p < 0.01 vs. indicated group

To further explore the relationship between Epac1 and SOCS3, naïve rats received SOCS3‐siRNA injection in PVN to mimic the neonatal‐CRD‐induced downregulation of SOCS3 expression. SOCS3 protein expression was significantly decreased in PVN subsequent to SOCS3‐siRNA injection in naïve rats (t [10] = 10.21, *p* = 0.01; Figure S5), as well as the nociceptive threshold (Figure 6A). Meanwhile, AWR scores were increased at 20 (*p* = 0.01), 30, 40 and 50 mmHg 36 h after SOCS3‐siRNA treatment (*p* < 0.01, Figure 6B), with the double immunofluorescence of c‐Fos protein and DAPI visualized (Figure 6C). The density of c‐Fos‐positive cells in PVN was significantly increased (t [10] = 9.40, *p* < 0.01; Figure 6D). Next, the potential relationship between inhibition of SOCS3 and activation of HPA axis was explored. Plasma levels of CRF, ACTH, and CORT were significantly increased 36 h after SOCS3‐siRNA administration compared with control rats (*p* = 0.04, *p* = 0.02, *p* = 0.03, respectively, Figure 6E–G). As aforementioned, downregulated Epac1‐SOCS3 pathway was consistently coupled with activated HPA axis in neonatal‐CRD‐induced chronic visceral hypersensitivity, which can be achieved by silencing of SOCS3 in naïve rats.

### 3.7 Functional duality of IL‐6 in visceral hypersensitivity in adult‐CRD and neonatal‐CRD rats

**FIGURE 7:**
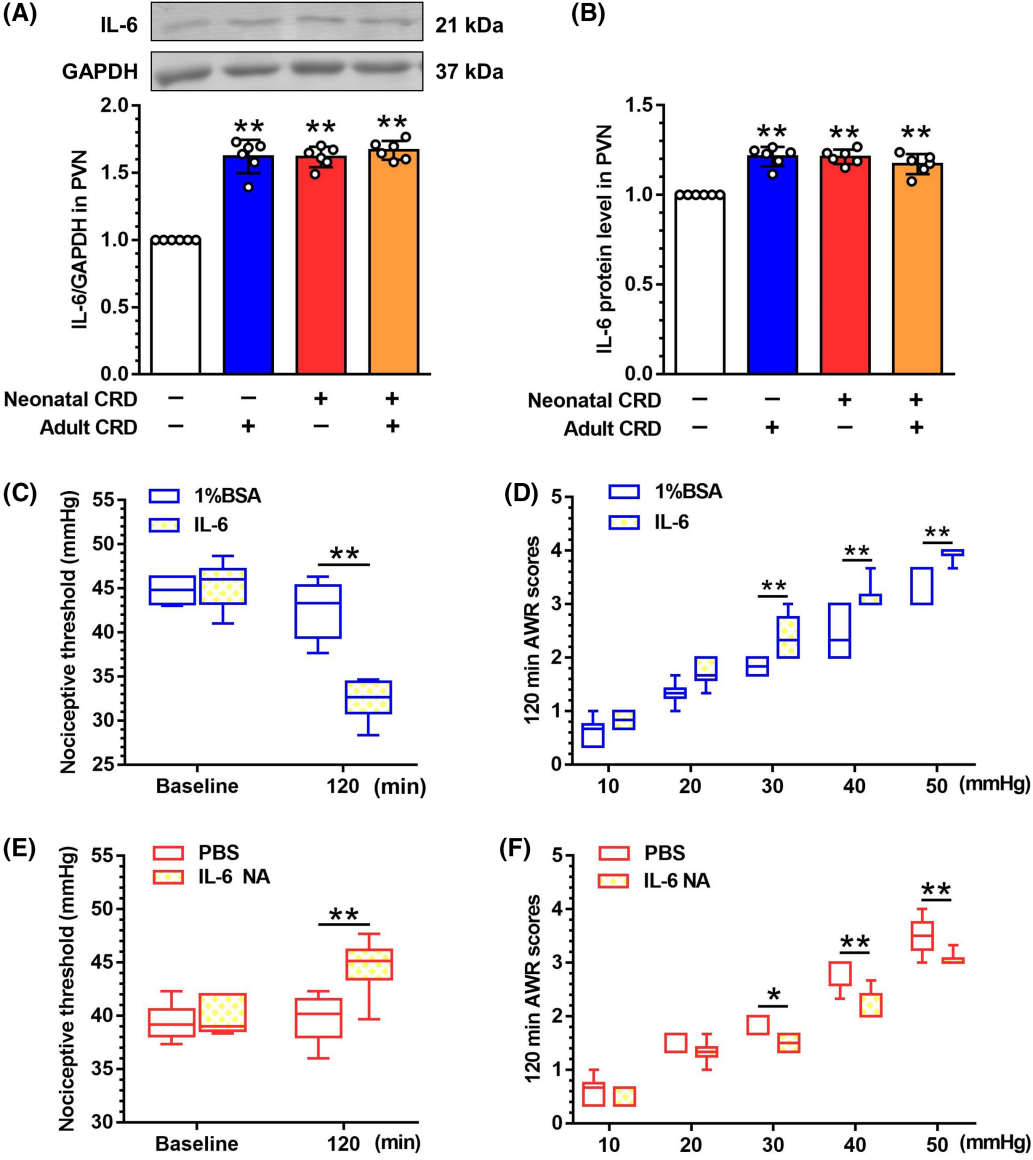
Involvement of IL‐6 in neonatal‐CRD‐induced chronic visceral hypersensitivity. (A) Western blotting indicated that IL‐6 protein expression was significantly upregulated in neonatal‐CRD, adult‐CRD, and dual‐CRD groups, respectively (*n* = 6). (B) ELISA indicated that IL‐6 protein expression was also significantly upregulated (*n* = 6). (C) The visceral nociceptive threshold decreased 120 min after intra‐PVN administration of exogenous IL‐6 in control group (*n* = 6). (D) The AWR scores were increased at 30, 40, and 50 mmHg 120 min after intra‐PVN administration of exogenous IL‐6 in control group (*n* = 6). (E) Nociceptive threshold increased 120 min after PVN administration of IL‐6 neutralizing antibody (NA) in neonatal‐CRD group (*n* = 6). (F) The AWR scores were decreased at 30, 40, and 50 mmHg 120 min after intra‐PVN administration of exogenous IL‐6 NA in neonatal‐CRD group (*n* = 6). Data are presented as the mean ± SD. *p < 0.05, **p < 0.01 vs. indicated group

The role of IL‐6 in the neonatal‐CRD‐induced chronic visceral pain was explored. Elevated IL‐6 levels in neonatal‐CRD, adult‐CRD, and dual‐CRD groups as compared to control group (F [3,20] = 93.74, *p* < 0.01, Figure 7A), as was also true of ELISA results (F [3,20] = 31.50, *p* < 0.01; Figure 7B). Accordingly, the nociceptive threshold was decreased significantly 2 h after intra‐PVN injection of exogenous IL‐6 in the naïve rats (*p* < 0.01, Figure 7C). AWR scores were increased at 30, 40, and 50 mmHg 120 min after IL‐6 treatment in naïve rats (*p* < 0.01, Figure 7D). In neonatal‐CRD rats, pharmacological intervention of IL‐6 with IL‐6 neutralizing antibody (NA) elevated nociceptive threshold 120 min after administration (*p* < 0.01, Figure 7E). AWR scores were decreased at 30 (*p* = 0.03), 40, and 50 mmHg (*p* < 0.01) 120 min after IL‐6 NA treatment in neonatal‐CRD rats (Figure 7F). In adult‐CRD rats, rats experiencing adult CRD showed no sign of recovery with respect to visceral nociceptive threshold as vehicle rats did otherwise at 120 min after pre‐administration with IL‐6 NA (*p* < 0.01, Figure S6). AWR scores were increased at 20, 30, 40, and 50 mmHg 120 min after CRD stimulation in naïve rats pretreated with IL‐6 NA (*p* < 0.05 or *p* < 0.01, Figure S7). Moreover, SOCS3 protein was downregulated at 120 min after CRD stimulation in the control rats pretreated with IL‐6 NA (t [8] = 6.05, *p* < 0.01; Figure S8). All the original, uncropped gel/blot images were provided in Appendix S1. Overall, these results indicated that neonatal‐CRD‐induced downregulated SOCS3 could not effectively inhibit the IL‐6‐induced inflammatory signaling in PVN in neonatal‐CRD group.

## 4 DISCUSSION

This study aimed to explore whether Epac1‐SOCS3 signaling in CRF neurons participates in neonatal‐CRD‐induced visceral hypersensitivity. We for the first time demonstrated that the expression and activity of Epac1 and its downstream effector SOCS3 in PVN were decreased in rats with neonatal‐CRD‐induced visceral hypersensitivity, which can be alleviated by Epac agonist 8‐pCPT or SOCS3 genetic overexpression. These results indicate that Epac1‐SOCS3 pathway contributes to the sensitization of CRF neurons in PVN and precipitates visceral hypersensitivity.

Noxious CRD, as a mechanical stimulatory stress in early life, has a potential, profound, and more serious effect on visceral sensation and sensitivity. In this study, we focused on the effects of early‐life stress on the visceral hypersensitivity. Moreover, the rat strain is more stable in visceral pain‐related behavioral detection. However, several other models have been simulated and validated for IBS from certain aspects of human symptoms. The role of psychological factors in functional disorders can be intensified by early‐life miserable events, such as maternal separation, maternal deprivation, and bedding reduction. Thus, we recruited the neonatal‐CRD rats to study the mechanical visceral hypersensitivity in IBS. Al‐Chaer and collaborators reported that neonatal colorectal insult induces adult visceral hypersensitivity long after the initial damage subsides. However, a dormant event can be triggered by a subthreshold stressor later in life. Our laboratory team has been engaged in the exploration of the mechanisms of visceral pain for over 20 years. We previously utilized dual distention at 80 mmHg (1 min) at a 5‐min interval to develop the adult CRD. Moreover, the visceral pain threshold of normal SD rats is generally below 60 mmHg, and 80 mmHg is a suprathreshold stimulus. Thereafter, we adopted the neonatal‐CRD paradigm, and adult‐CRD procedures were modified from the previous studies.^15^ The modified paradigm of 60 mmHg distension for 60 s, 10 times at a 15 s interval, was confirmed to be more stable and reasonable, which suffices to induce visceral hypersensitivity. We are justified that we have ameliorated the visceral hypersensitivity model and optimized the regimens.

Downregulation of Epac1 participates in hypersensitivity of CRF neurons in PVN in rats that experienced neonatal CRD. A paucity of studies has demonstrated that Epac1 is involved in the inflammation‐induced nociception. With pre‐clinical neuropathic or chronic pain model, Epac1 facilitated the hyperalgesia via multiple signaling pathways, for example, Rap1, PLC, PLD, MEK/ERK, GRK2, PKCα, and PKCε.^19^, ^20^, ^44^, ^45^, ^46^, ^47^ However, most of these studies only explore the Epac mechanism in pain on peripheral tissues or organs, especially dorsal root ganglion. The role of Epac signaling in brain on pain remains elusive. In our visceral hypersensitivity model, Epac1 levels in PVN were decreased. ESI‐09 is a competitive Epac inhibitor with a selectivity 100 times that of PKA.^48^ Intra‐PVN microinfusion of ESI‐09 or Epac1‐siRNA facilitated the visceral hypersensitivity and pain precipitation in control rats. Conversely, intra‐PVN administration of the Epac‐specific cAMP analog 8‐pCPT inhibited visceral nociceptive hypersensitivity in neonatal‐CRD rats. 8‐pCPT is a potent activator of Epac1, without affinity for Epac2.^49^ Notably, 15 min after administration of ESI‐09 or 8‐pCPT, visceral sensitivity was altered vs. baseline. In vitro, the firing rates of PVN CRF‐specific neurons were increased after ESI‐09 perfusion of the brain slices from control rats, in contrast to the decreased firing after 8‐pCPT irrigation of the brain slices from neonatal rats. These results authenticated the hypothesis that Epac1 is implicated in the visceral pain.

There is a paucity of studies available with respect to the difference between Epac signaling and PKA signaling in the brain in mediating the biological response to cAMP. In view of the importance of Epac1 in limiting the pro‐inflammatory IL‐6 signaling, the induction of SOCS3 via Epac‐dependent pathway might be varied in different cell systems. Yarwood et al. demonstrated that the anti‐inflammatory SOCS3 involving activation of CCAAT/enhancer‐binding proteins (C/EBPs) was induced by Epac1 and the Rap1 in COS1 cells and human umbilical vein endothelial cells (HUVECs).^50^, ^51^ The induction of SOCS3 and subsequent inhibition of cytokine‐mediated activation of ERK1,2 and STAT3 in response to cAMP were controlled by Epac1 and a PKA‐independent activation of ERK pathway.^52^ SOCS3 might also be induced via Epac‐regulated JNK activity in HUVBECs and other cell types.^53^ Accordingly, the remaining signaling processes accounting for SOCS3 induction invite further elucidation. Herein, downregulation of Rap1 and SOCS3 mediates chronic visceral hypersensitivity induced by neonatal CRD. Epac1 agonist 8‐pCPT stimuli induce the activation of local Rap1 pool in cells,^54^, ^55^ and it has been demonstrated that activates Rap signal, such as PLC‐ε, PKC, and MAPK in vitro.^21^, ^56^ As downstream targets of Epac1, Rap1 and SOCS3 in PVN were also decreased in rats with visceral hypersensitivity. Local inhibition of PVN Epac1 or SOCS3 in naïve rats produced visceral hypersensitivity. Silencing of SOCS3 by RNA interference also yielded the same results as Epac1‐siRNA in naïve rats, that is, reduced visceral pain threshold, elevated c‐Fos immunoreactivity in PVN, and activated HPA axis. These results demonstrated that Epac1/SOCS3 pathway is indeed associated with the adaptive protection to control visceral pain‐related diseases. Noxious visceral irritation is well acknowledged to induce the expression of c‐Fos in PVN.^57^ Additionally, upregulation of SOCS3 via intra‐PVN microinjection of AAV‐SOCS3 reversed visceral hypersensitivity. The above findings confirmed the crucial role of SOCS3 in the visceral pain.

Upregulation of SOCS3 in PVN reversed the neonatal‐CRD‐induced chronic visceral hypersensitivity, whereas downregulation of SOCS3 contributed to disinhibition of IL‐6 signaling and mediation of neonatal‐CRD‐induced chronic visceral pain. The pro‐inflammatory effect of elevated IL‐6 expression has been evidenced in the plasma from IBS patients and brain tissues from ELS rats.^58^, ^59^, ^60^ Gut inflammation evokes significant modification of brain‐gut axis, which is often alluded to as the persistent neuroinflammation. SOCS3 is considered protective against neuroinflammation by inhibition of IL‐6 receptor‐mediated signaling.^61^ IL‐6 possesses both pro‐ and anti‐inflammatory effects. Despite its controversial inflammatory properties with SOCS3, studies have identified that IL‐6 promotes acute and chronic inflammatory diseases in the absence of SOCS3.^62^ IL‐6 can reportedly upregulate SOCS3 expression, which in turn downregulate the expression of CRF gene in hypothalamic cells.^63^ Intriguingly, the dichotomy in function of IL‐6 and SOCS3 was identified. Normal adult rats were subjected to CRD, and 2 h thereafter were termed as adult‐CRD rats. Despite the restored visceral pain threshold in the adult‐CRD rats, expression levels of IL‐6, Epac1, and SOCS3 were still elevated, suggesting that anti‐inflammatory signaling in the adult‐CRD rats was rapidly aroused by the induction of SOCS3 through dual triggers of Epac1 and IL‐6. However, IL‐6 NA administration in PVN rendered the visceral imbalance in adult‐CRD rats, implying that the intracellular SOCS3 did not respond potently owing to the collapse of negative feedback loop evoked by IL‐6. In neonatal‐CRD or dual‐CRD rats, various pro‐inflammatory cytokines, including IL‐6, IL‐1β, and TNF‐α, contribute to the sensitization of CRF neurons in PVN. The long‐lasting activation of the PVN CRF neurons may cause changes in neuronal plasticity. In addition, the downregulation of anti‐inflammatory signaling involving Epac1/SOCS3 exacerbated the sensitized condition, thereby inducing both hormonal and behavioral modification. Furthermore, IL‐6 NA diminished the nociception in rats that experienced neonatal CRD, indicative of the sustained pro‐inflammatory effect of IL‐6 in chronic visceral pain. In addition, intra‐PVN administration of exogenous IL‐6 in the naïve rats simulated the visceral hypersensitivity. These results were consistent with the finding of Epac1‐SOCS3 pathway in inhibition of pro‐inflammatory effect with respect to IL‐6 signaling.^53^, ^64^, ^65^, ^66^

**FIGURE 8:**
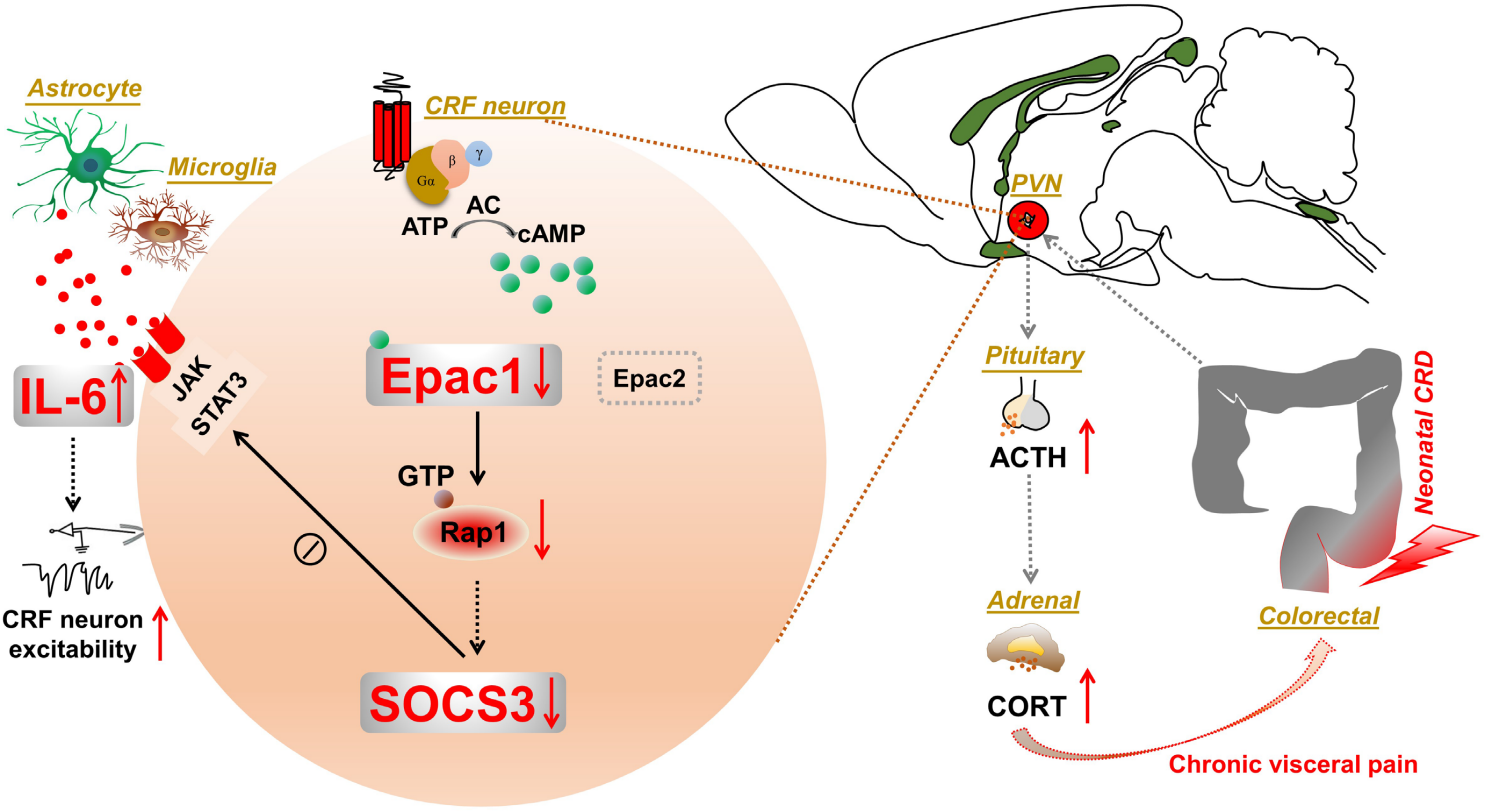
Schema of the experiment. In neonatal‐CRD‐induced visceral hypersensitivity, both Epac1 and SOCS3 were downregulated, resulting in the disinhibition of IL‐6 receptor‐mediated signaling and amplification of IL‐6 pro‐inflammatory signals and further activation of CRF neurons in PVN and the HPA axis. Activation of either Epac1 or SOCS3, or antagonism on IL‐6, reversed the visceral nociceptive effects in the neonatal‐CRD group. The upward arrow represents upregulation and the downward arrow stands for downregulation

Together, our study proposed a notion that early‐life stress disrupts the Epac1/Rap1 pathway and subsequently SOCS3 (Figure 8), which triggers sensitized CRF neurons in PVN and amplified IL‐6 signaling in neuroinflammation and visceral nociception. This is a pilot study of the molecular mechanism addressing Epac1‐SOCS3 effects in PVN in neonatal‐CRD‐induced chronic visceral hypersensitivity, and we affirmed the association of Epac1‐SOCS3 pathway with the anti‐inflammatory effect, which is also supported by the reported anti‐inflammatory role of Epac1‐SOCS3 pathway particularly in vascular endothelial cells.^64^ From a translational perspective, our study might contribute a new avenue for the development of new treatment on disorders with visceral hypersensitivity and pain.

## AUTHOR CONTRIBUTION

YMZ conceived of the study. STH and BBC carried out the experiments, performed the statistical analyses for molecular and behavioral measures, and participated in the coordination of the study. YMZ and STH drafted the manuscript. ZJS completed the electrophysiological experiments and performed the statistical analysis. HLT assisted in the experimental process. RH contributed to the final version. All authors read and approved the final manuscript.

## CONFLICTS OF INTEREST

The authors declared no conflict of interests.

## Supporting information


Figure S1

Figure S2

Figure S3

Figure S4

Figure S5

Figure S6

Figure S7

Figure S8

Appendix S1

## Data Availability

Data are available upon request.
