# Supplementary figures and images for: Unraveling the role of Epac1‐SOCS3 signaling in the development of neonatal‐CRD‐induced visceral hypersensitivity in rats
Source: CNS Neurosci Ther. 2022 Jun 15;28(9):1393–408. doi: 10.1111/cns.13880 (PMC9344090; doi:10.1111/cns.13880)

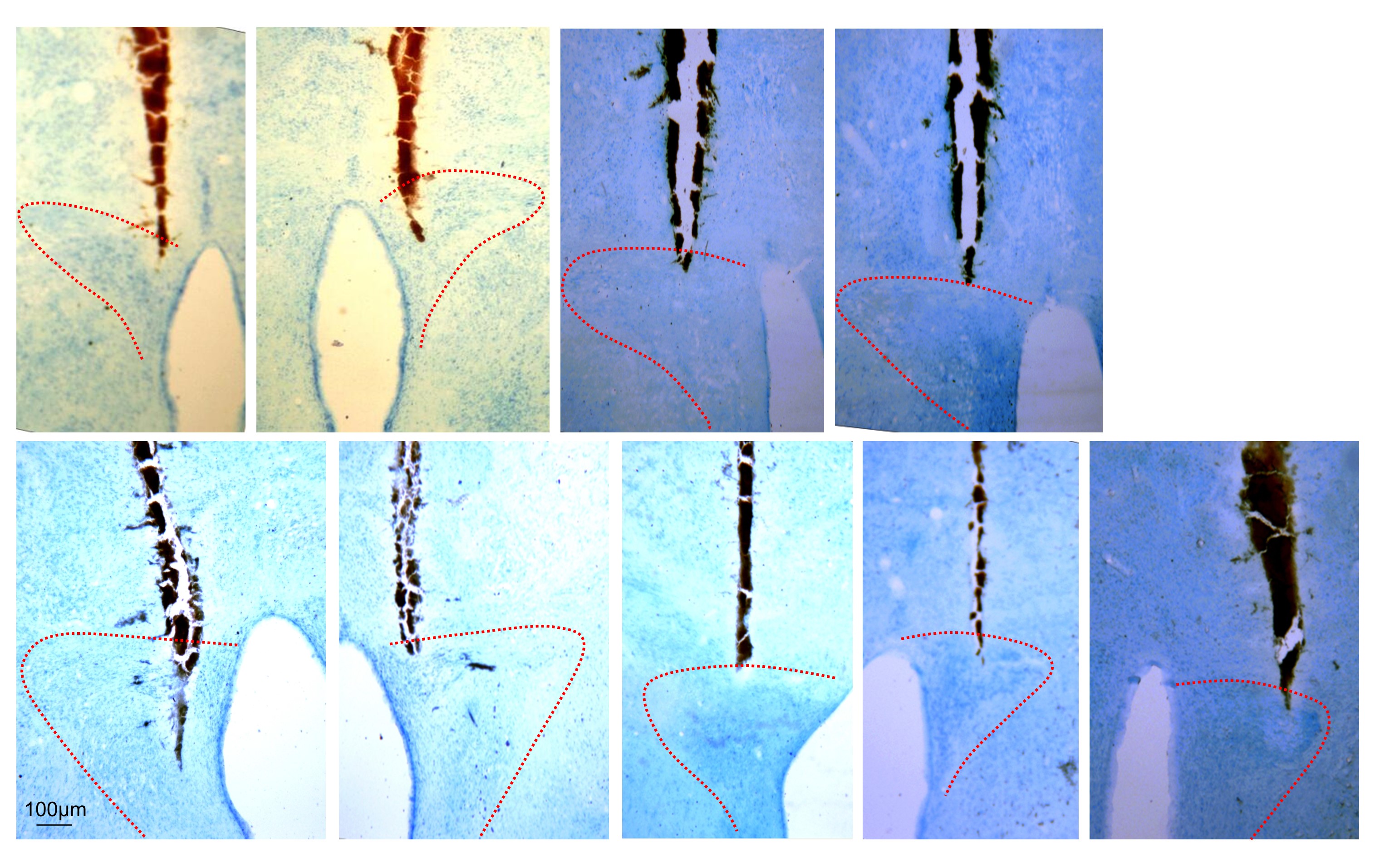

Supplement: Supplementary file 1 — Figure S1 [file CNS-28-1393-s008.jpg]

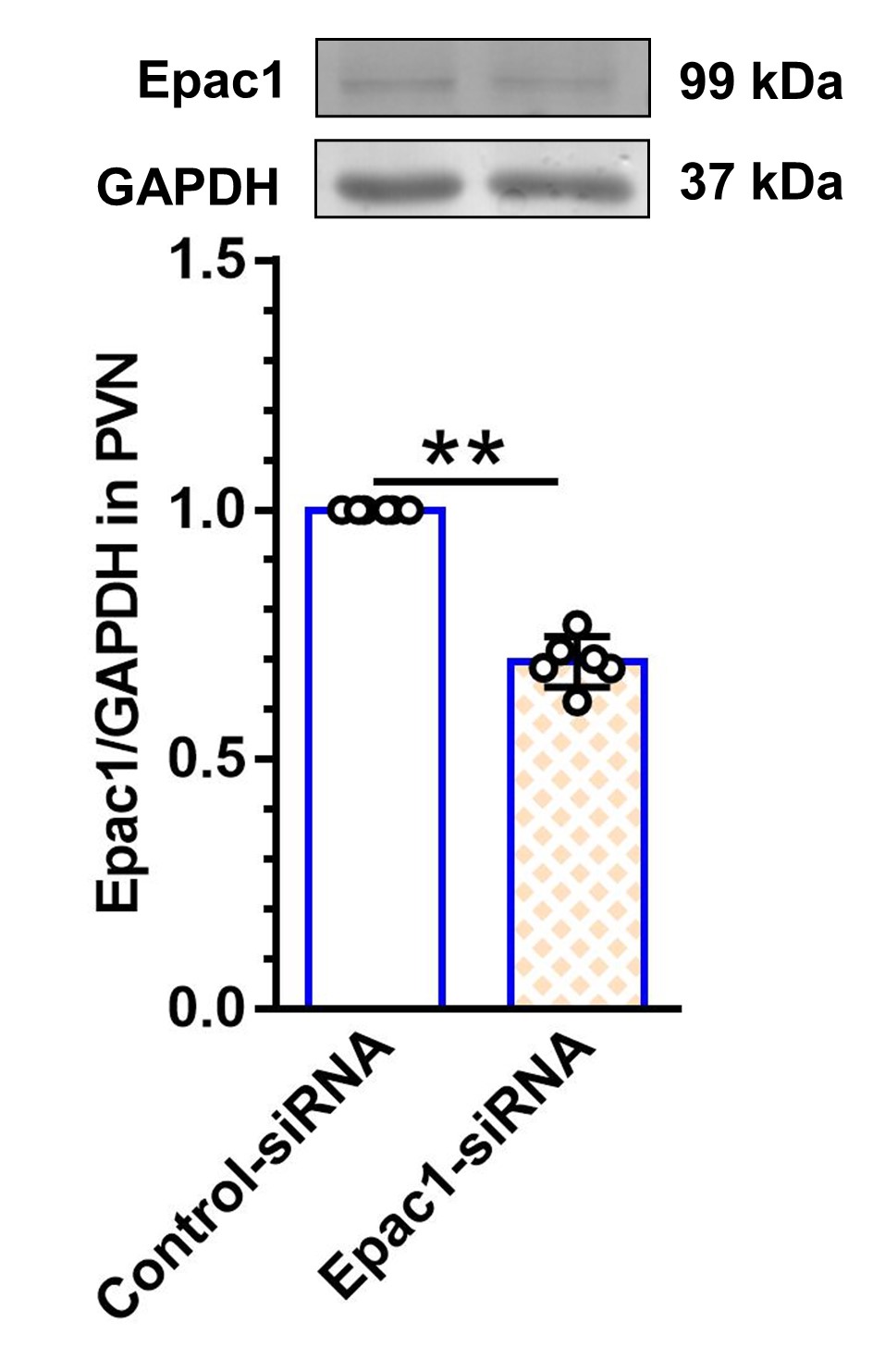

Supplement: Supplementary file 2 — Figure S2 [file CNS-28-1393-s003.jpg]

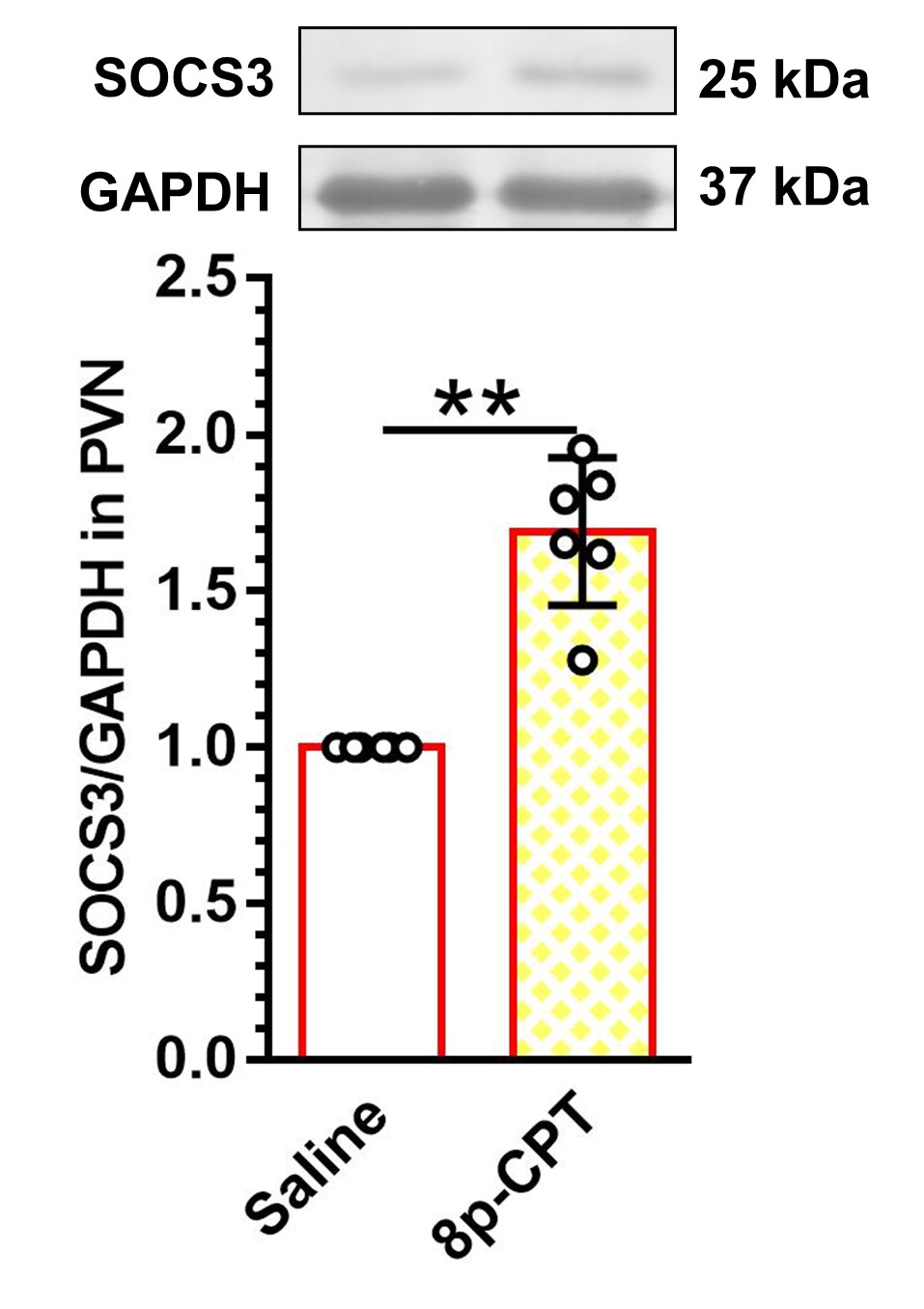

Supplement: Supplementary file 3 — Figure S3 [file CNS-28-1393-s006.jpg]

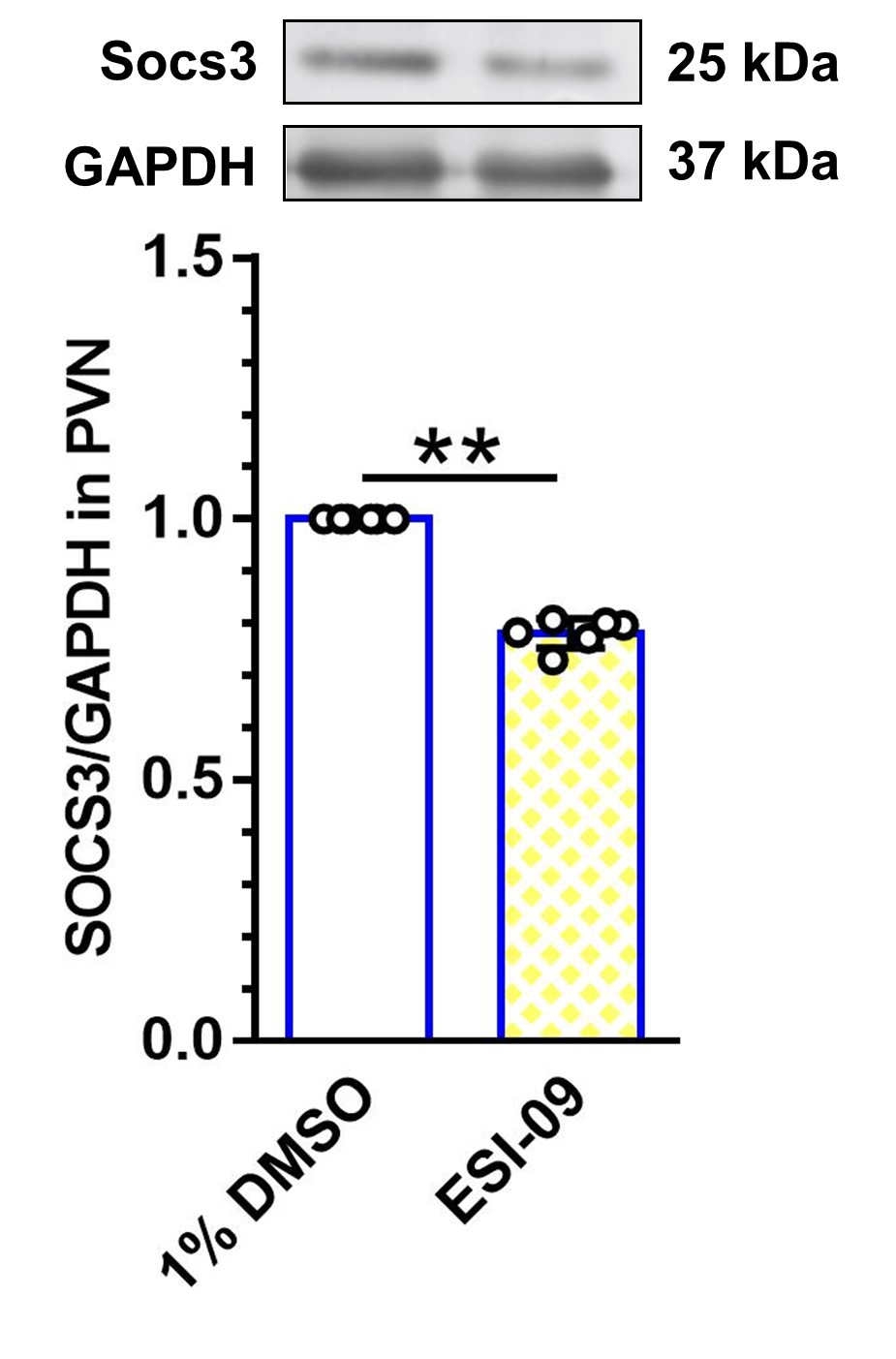

Supplement: Supplementary file 4 — Figure S4 [file CNS-28-1393-s002.jpg]

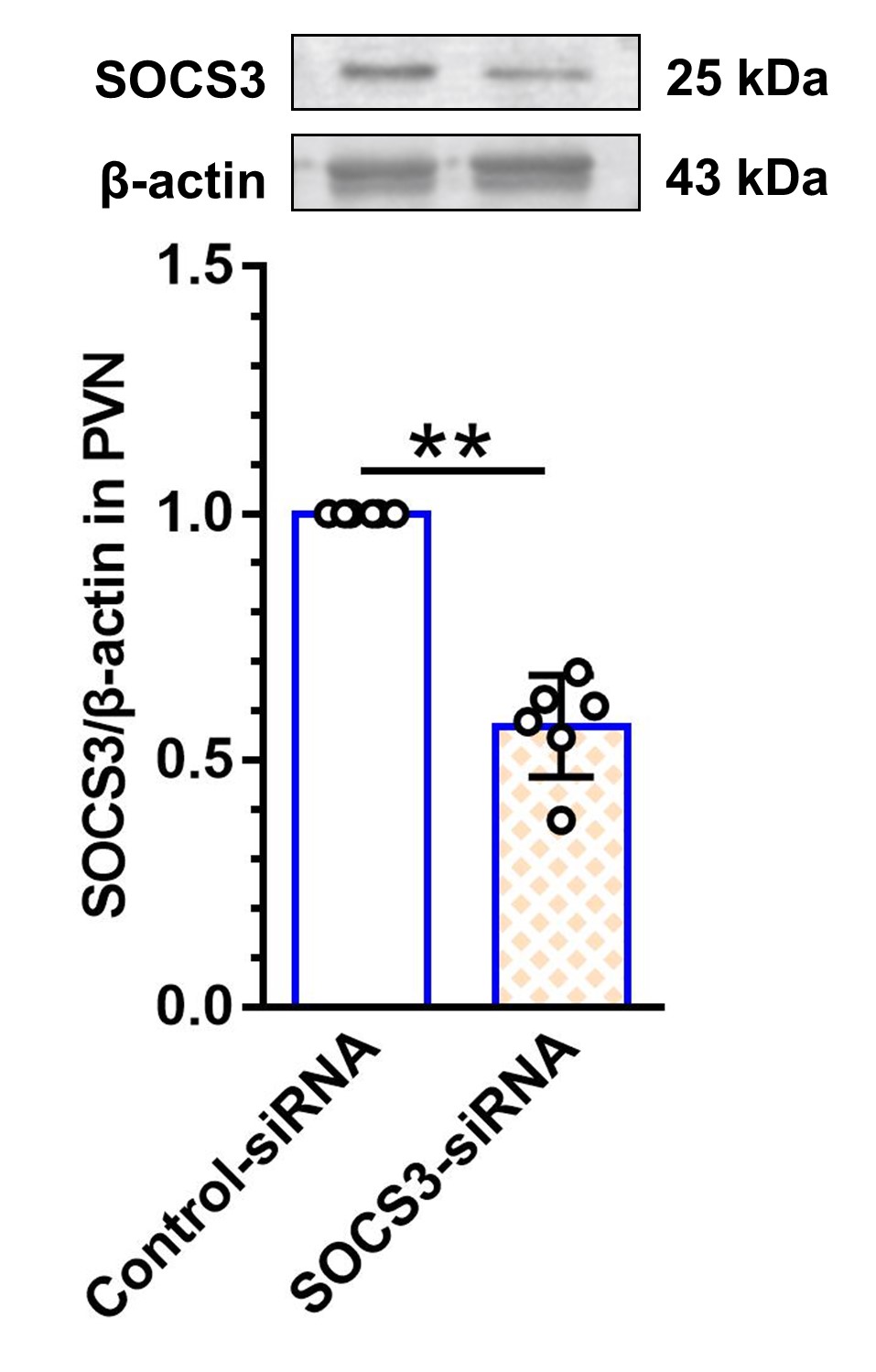

Supplement: Supplementary file 5 — Figure S5 [file CNS-28-1393-s007.jpg]

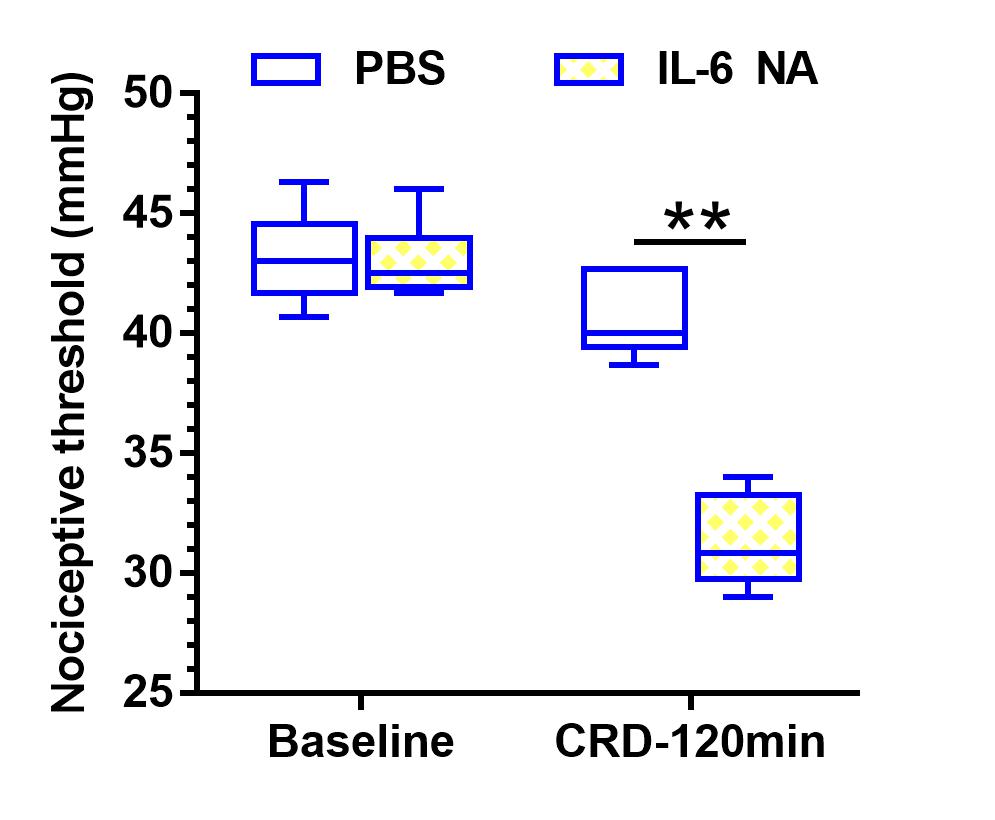

Supplement: Supplementary file 6 — Figure S6 [file CNS-28-1393-s004.jpg]

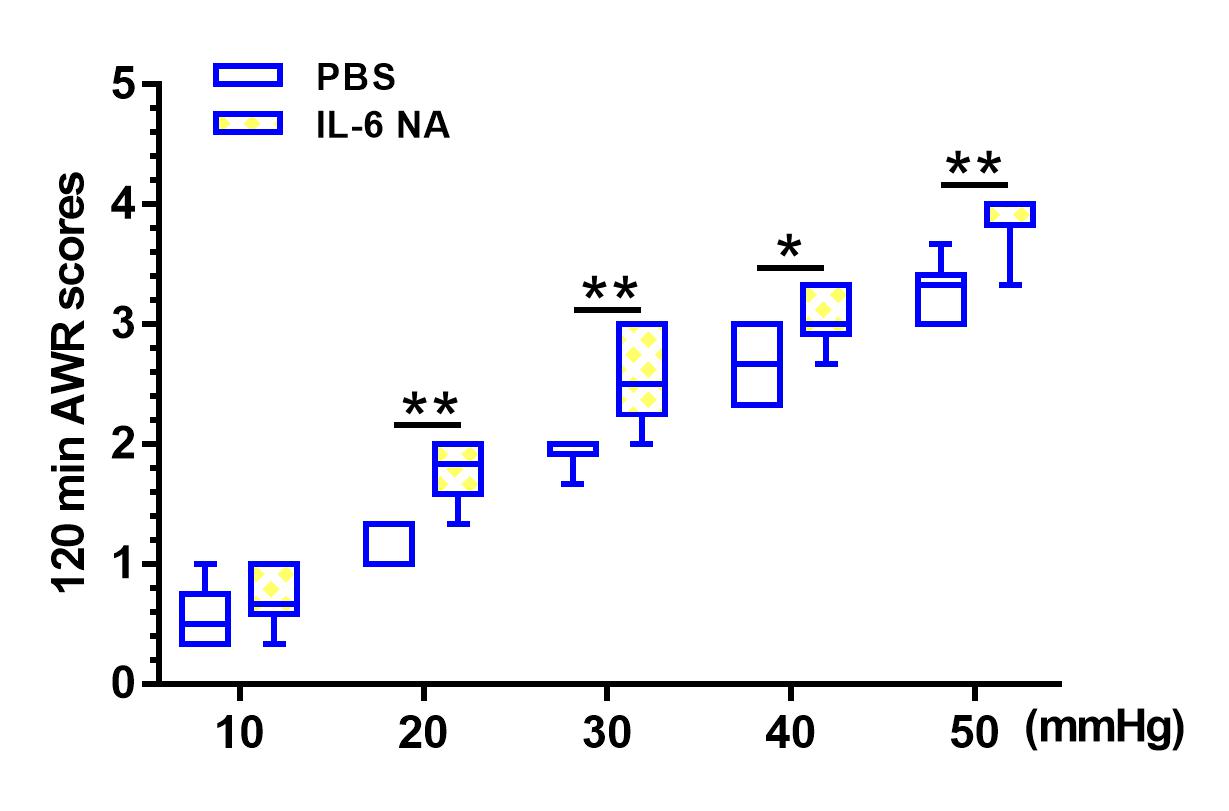

Supplement: Supplementary file 7 — Figure S7 [file CNS-28-1393-s001.jpg]

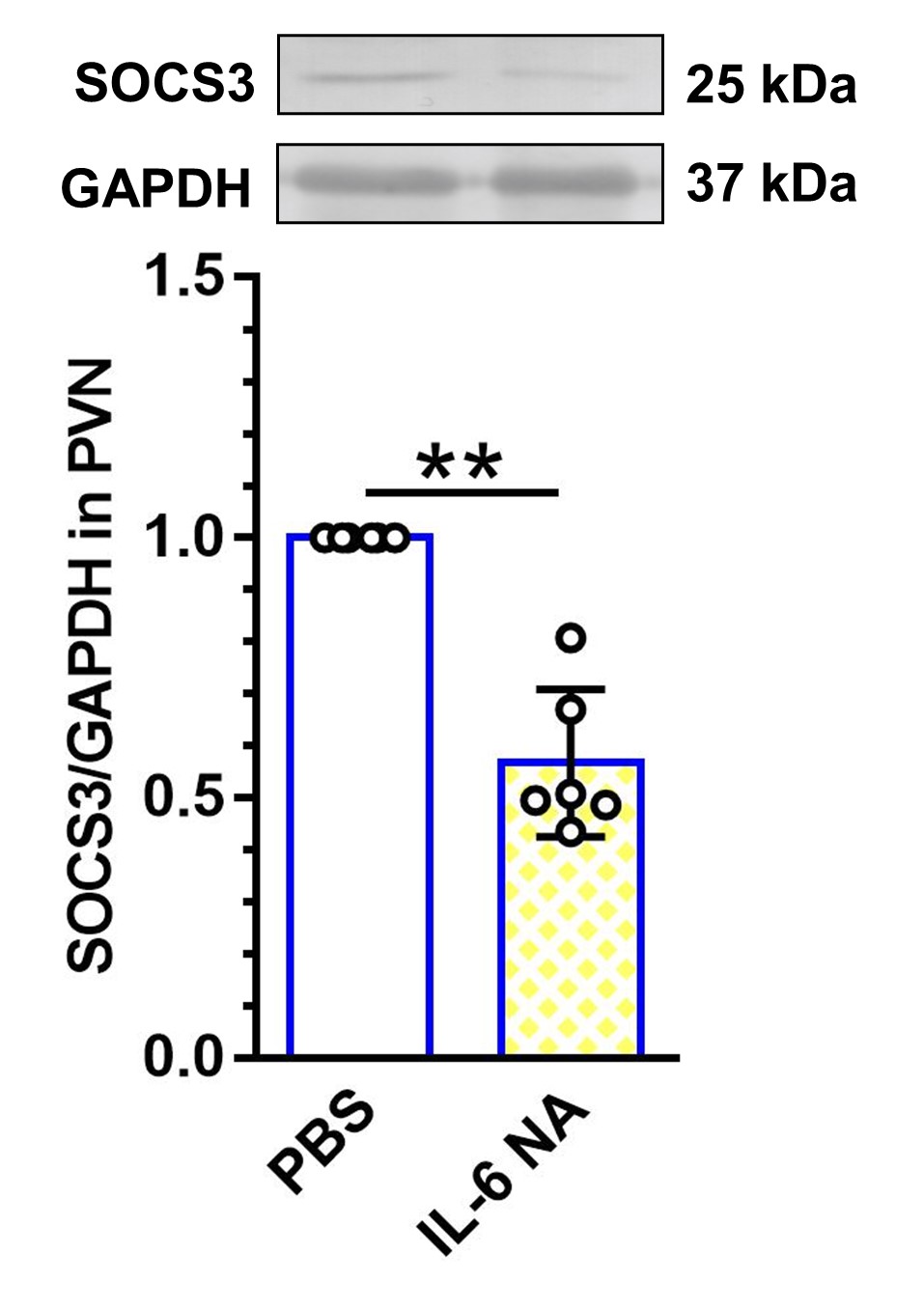

Supplement: Supplementary file 8 — Figure S8 [file CNS-28-1393-s009.jpg]
