# Supplementary material for: Unraveling the role of Epac1‐SOCS3 signaling in the development of neonatal‐CRD‐induced visceral hypersensitivity in rats
Source: CNS Neurosci Ther. 2022 Jun 15;28(9):1393–408. doi: 10.1111/cns.13880 (PMC9344090; doi:10.1111/cns.13880)

In this supplemental file, we would attach the original, uncropped gel/blot images appearing in the manuscript. Details are as follows:

Full unedited gel/blot for Figures

Figure 1

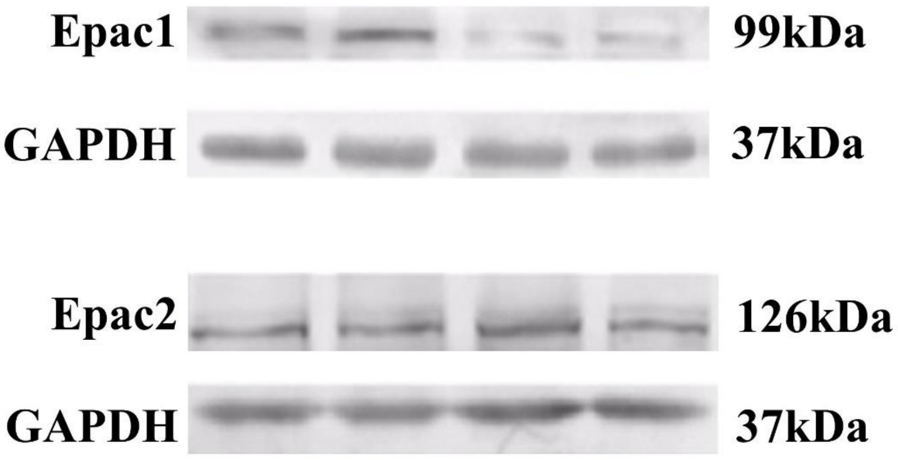

Figure 3

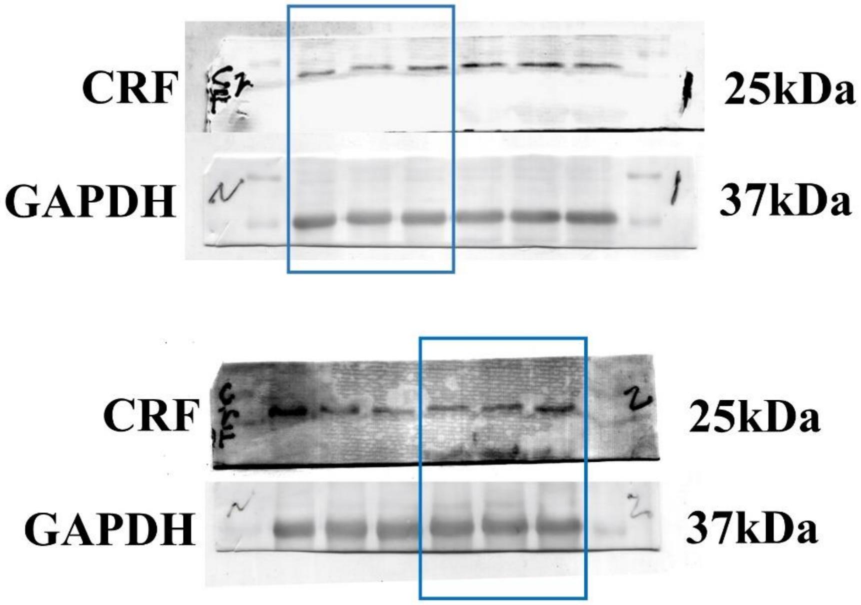

Figure 4

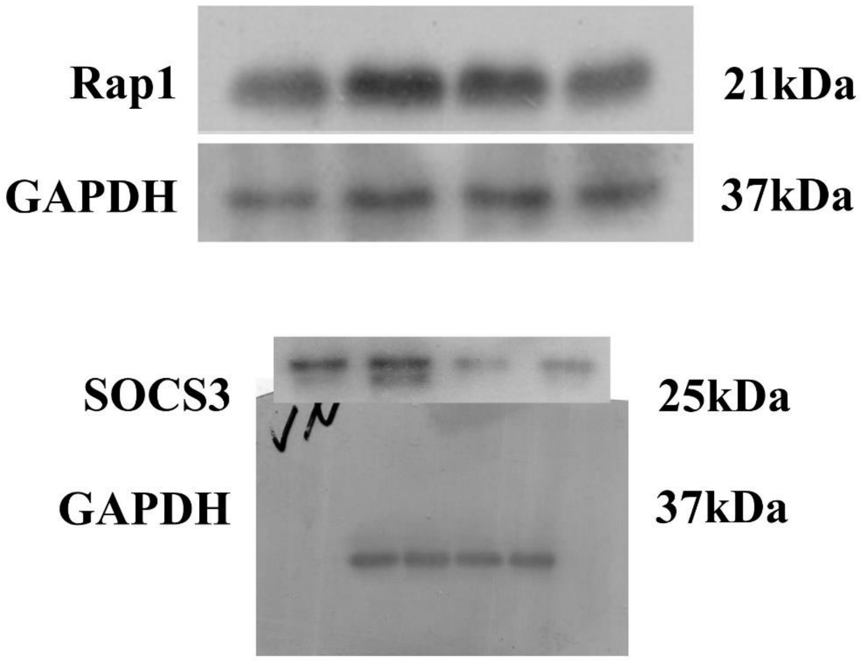

Figure 5

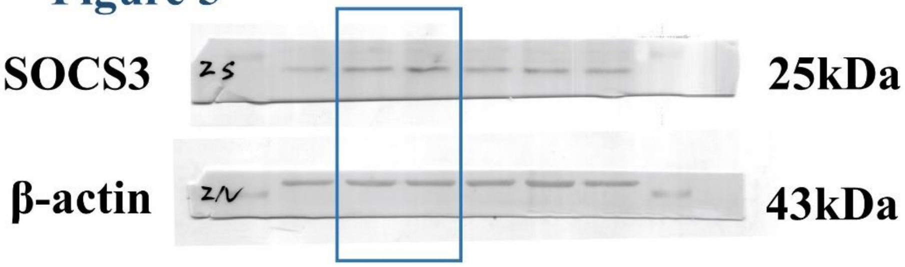

Figure 7

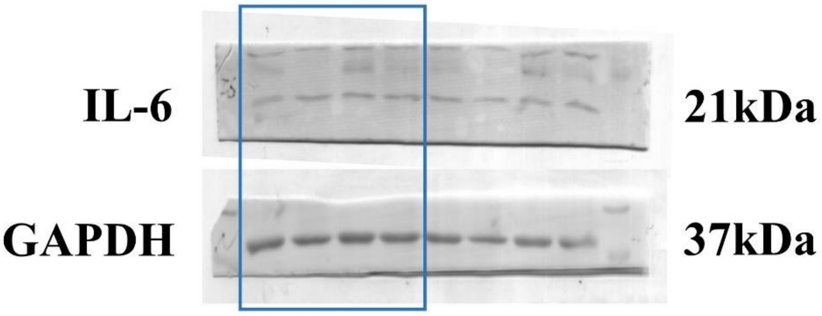

Figure S2

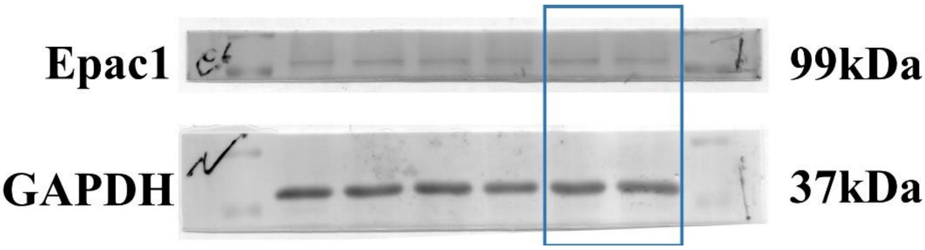

Figure S3

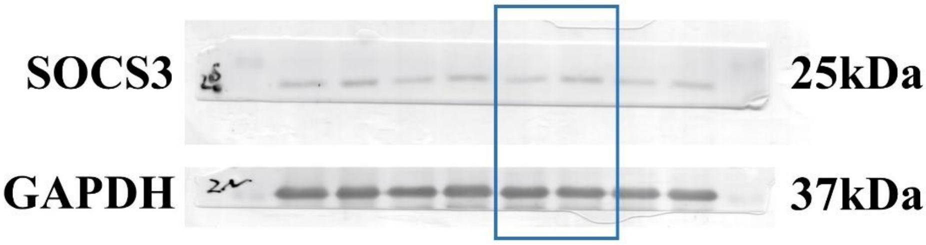

Figure S4

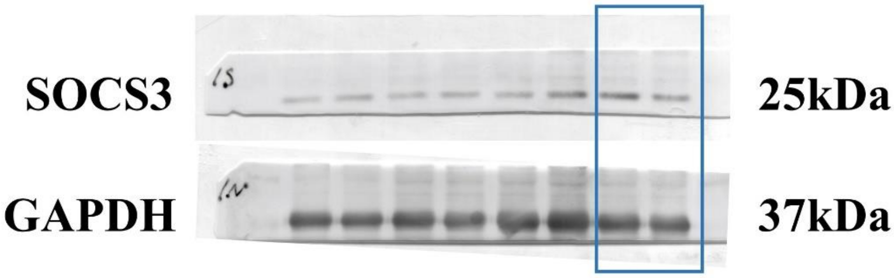

Figure S5

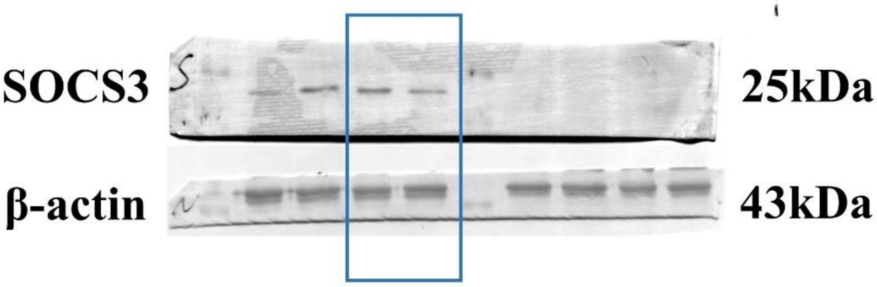

Figure S8

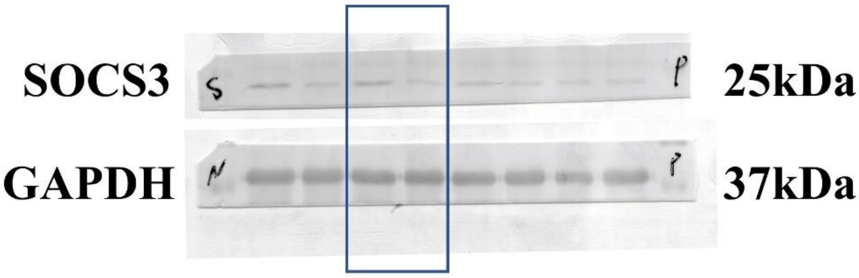

Supplement: Supplementary file 9 — Appendix S1 [file CNS-28-1393-s005.pdf]
